# Cdon acts as a Hedgehog decoy receptor during proximal-distal patterning of the optic vesicle

**DOI:** 10.1038/ncomms5272

**Published:** 2014-07-08

**Authors:** Marcos Julián Cardozo, Luisa Sánchez-Arrones, África Sandonis, Cristina Sánchez-Camacho, Gaia Gestri, Stephen W. Wilson, Isabel Guerrero, Paola Bovolenta

**Affiliations:** 1 Centro de Biología Molecular Severo Ochoa, Consejo Superior de Investigaciones Científicas—Universidad Autónoma de Madrid, 28049 Madrid Spain; 2 Ciber de Enfermedades Raras (CIBERER), ISCIII, c/ Nicolas Cabrera 1, 28049 Madrid Spain; 3 Department of Cell and Developmental Biology, University College London, Gower Street, London WC1E 6BT, UK; 4 Present address: Universidad Europea, C/ Tajo, s/n Villaviciosa de Odón, 28670 Madrid, Spain

## Abstract

Patterning of the vertebrate optic vesicle into proximal/optic stalk and distal/neural retina involves midline-derived Hedgehog (Hh) signalling, which promotes stalk specification. In the absence of Hh signalling, the stalks are not specified, causing cyclopia. Recent studies showed that the cell adhesion molecule Cdon forms a heteromeric complex with the Hh receptor Patched 1 (Ptc1). This receptor complex binds Hh and enhances signalling activation, indicating that Cdon positively regulates the pathway. Here we show that in the developing zebrafish and chick optic vesicle, in which *cdon* and *ptc1* are expressed with a complementary pattern, Cdon acts as a negative Hh signalling regulator. Cdon predominantly localizes to the basolateral side of neuroepithelial cells, promotes the enlargement of the neuroepithelial basal end-foot and traps Hh protein, thereby limiting its dispersion. This Ptc-independent function protects the retinal primordium from Hh activity, defines the stalk/retina boundary and thus the correct proximo-distal patterning of the eye.

## Abstract (web summary)

The *Drosophila* homologue of the vertebrate cell surface glycoprotein Cdon binds Hedgehog ligand and thereby prevents its diffusion. Here, the authors provide evidence for a similar mechanism during vertebrate optic vesicle patterning, where Cdon acts as a negative regulator of Hedgehog signalling to define the boundary between the optic stalk and the retina.

## Graphical abstract

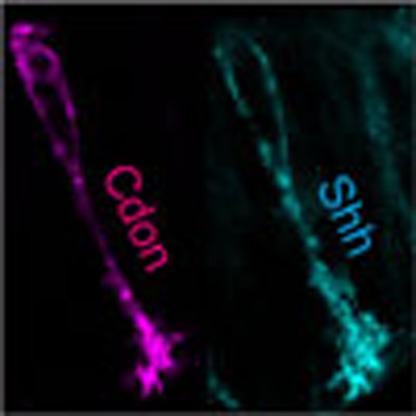

The Hedgehog (Hh) signalling pathway is a highly conserved regulator of patterning and homeostatic events in various developing and adult organs and thus its activation is under tight control^1^,^2^. Hh ligands are released through the cooperative activity of the transmembrane protein Dispatched and heparan sulphate proteoglycans, which are also crucial to modulate long-range distribution^3^,^4^,^5^. There is also evidence that cytonemes, long filopodial-like extensions formed by the producing cells, promote delivery of Hh-containing vesicles to similar extensions in target cells^6^,^7^,^8^. In the targets, binding of Hh to a receptor complex containing the seven-pass membrane protein Patched (Ptc), releases Ptc-mediated inhibition of the signal transducer protein Smoothened (Smo), which initiates a cascade of intracellular events that lead to the transcription of Hh target genes^9^,^10^.

Cdon (cell adhesion molecule-related, downregulated by oncogenes)^11^,^12^, the related Boc (Brother of Cdo)^13^ or their *Drosophila* counterparts Ihog (Interference hedgehog) and Boi (Brother of Ihog), are additional components of the Hh receptor complex^14^. Cdon and Boc are cell surface glycoproteins that belong to a subgroup of the immunoglobulin (Ig) super-family of cell adhesion molecules^14^. Their ectodomains contain five and four immunoglobulin-like domains, respectively, followed by three fibronectin III (FNIII) repeats (1–3), which are involved in Ptc and Hh binding. FnIII(1-2) domains interact with Ptc^15^, whereas the evolutionary conserved FnIII(3) domain binds Hh with high affinity^16^,^17^,^18^.

The requirement of a heteromeric interaction between Ptc and Ihog/Boi for both high-affinity Hh binding and presentation of Ptc at the cell surface was initially demonstrated in *Drosophila*^19^ and then shown to be conserved in vertebrates^15^. In mice, Cdon and Boc and the unrelated Hh binding protein Gas1 (refs 20, 21) act as positive and redundant regulators of Hh signalling in different contexts^22^,^23^,^24^. For example, *Cdon*-deficient mice are characterized by mild holoprosencephalia (HPE) with microphthalmia and moderate facial and brain defects of strain specific penetrance^11^,^25^,^26^,^27^. These traits are aggravated by the additional inactivation of *Boc*, so that *Cdon*^−/−^;*Boc*^−/−^ embryos present a significant loss of the Hh pathway activity^24^, which is further worsened by *Gas1* inactivation. Indeed *Cdon*^−/−^;*Boc*^−/−^;*Gas1*^−/−^ compound mutant embryos show a phenotype similar to that caused by the loss of Sonic hedgehog (Shh) function: strong facial malformations and failure of partition of the cerebral hemispheres and eye primordia^22^. In zebrafish, analysis of the *uml(boc)* mutant supports the idea that Boc acts as a positive regulator of Hh signalling in the ventral neural tube but might have an opposite function in the lower jaw^28^.

In vertebrates, the morphogenesis of the eye primordium, also known as optic vesicle, occurs concomitant to its patterning along the proximal-distal (P-D) axis in medial (prospective optic stalk) and lateral (prospective retinal) domains. The establishment of this early P-D patterning is defined by the expression of two pair- and homeobox- containing transcription factors, *Pax2* and *Pax6*, the expression of which is, respectively, restricted to the optic stalk and the retinal primordium^29^,^30^,^31^. Functional studies have demonstrated that Hh secreted from the midline of the ventral forebrain is required to promote *Pax2* expression and thus to establish optic stalk identity^30^,^32^,^33^. A cross-repressing regulatory loop between Pax2 and Pax6 then refines the pattern, forming a precise boundary between the retina and the optic stalk^29^. In the absence of Hh signalling, the stalk does not form and Pax6-positive vesicles do not separate, forming a single cyclopic eye^33^.

Notably, in the optic vesicles *ptc* and *cdon* are distributed with a complementary and evolutionary conserved pattern: Cdon localizes to the distal region overlapping with Pax6 expression, whereas *ptc1* matches Pax2 distribution^26^,^34^,^35^ (Fig. 1f–i; Supplementary Fig. 1). Here we have addressed whether and how *cdon* contributes to optic vesicle patterning, using zebrafish and chick embryos as model systems. Our results indicate that in the eye, Cdon acts as a negative regulator of Hh signalling. Cdon as well as Boc expand the surface of the basal end-foot of neuroepithelial cells, bind Hh and prevent its dispersion, thus limiting long-range signalling, as proposed for Ihog and Boi^19^,^36^,^37^,^38^. This function protects the retinal primordium from Hh activity, thus enabling a correct P-D patterning of the eye.

## Results

### *cdon* and *ptc* localize to complementary optic vesicle domains

**Figure 1:**
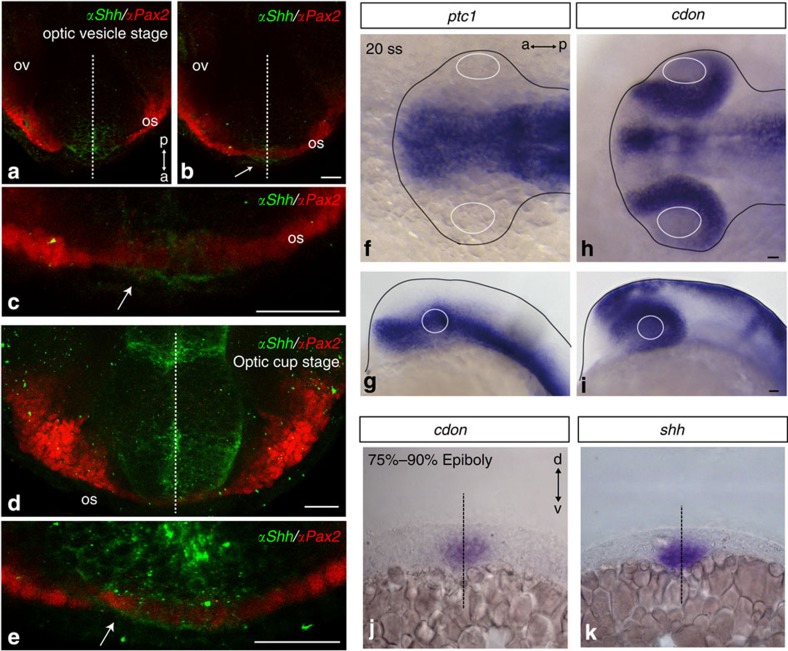
Expression of *cdon* and other Hh signalling components during P-D patterning of the optic vesicles. (**a**–**c**) Ventral views of anterior zebrafish forebrain at optic vesicle (**a** is ventral to **b**) and optic cup (**d**, **e**) stages immunostained *in toto* with antibodies against Pax2 and Shh. (**f**–**i**) Dorsal (**f**,**h**) and lateral (**g**,**i**) views of zebrafish embryos hybridized *in toto* with probes specific for *ptc1* and *cdon* at the 20 somites’ stage (20 ss). (**j**–**k**) Coronal sections of embryos (75–90% epiboly) hybridized for *cdon* and *shh*. The white arrows in **b**,**c** and **e** point to Shh immunolabelling in Pax2-positive cells. Dashed lines in **a**–**d**,**j** and **k** indicate the embryonic midline. Continuous white and black lines in **f**–**i** outline the lens and the embryonic border, respectively. a, anterior; d, dorsal; os, optic stalk; ov, optic vesicle; p, posterior; v, ventral. Scale bar, 25 μm.

*Pax2* function is critical to establish optic stalk identity in different vertebrate species^32^,^33^,^39^ and to restrain Pax6 expression, so that the two genes have a complementary expression restricted to the proximal/stalk and distal/retinal optic vesicle, respectively^29^,^32^ (Supplementary Fig. 1). Pax2 expression is regulated by Hh, which is strongly expressed in the ventral midline^30^,^32^,^33^. To get a better understanding of the role of Hh signalling in the P-D patterning of the eye, we analysed the distribution of Hh in zebrafish embryos. Hh was strongly localized to cells of the ventral midline of the forebrain and in optic stalk cells (Fig. 1a–e). At early stages of optic vesicle specification, Hh strongly accumulated at the most ventrally positioned Pax2-positive cells (Fig. 1c) but its distribution extended dorsolaterally as development progressed (Fig. 1d,e). In agreement with this distribution, the Hh receptor *ptc1* was expressed in the ventral forebrain including the prospective optic stalk^34^,^40^ (Fig. 1f,g). In contrast, *cdon*, but not the related *boc*^28^ (Supplementary Fig. 1w–z), was strongly localized to the neural retina region (Fig. 1h,i). This distribution was conserved in chick and mouse embryos (Supplementary Fig. 1k–o). In the latter, Cdon protein appeared to accumulate at the basolateral membrane of the retinal epithelium starting from E10, resembling the subcellular localization described for the *Drosophila* homologue Ihog^4^,^36^ (Supplementary Fig. 1t–v).

### *cdon* knockdown alters P-D patterning of the eye primordium

The complementary pattern of *ptc1* and *cdon* expression was consistent with the idea that, in the eye, the two proteins could act independently without forming a heteromeric Hh receptor^15^, raising the question of whether and how *cdon* expression in the retina contributes to eye development. Answering these questions, however, requires local or partial interference with Cdon function because *shh* colocalizes with *cdon* at the axial midline of gastrulating embryos (Fig. 1f,g). To avoid the complete abrogation of this early function that may be responsible for the mild holoprosencephalic phenotype of *Cdon* null mouse embryos^11^, we reduced *cdon* expression levels in zebrafish embryos using specific morpholinos (MOs).

Doses of 160 μM of a MO that efficiently interfered with the *cdon* translation start site (*Cdon*^*ATG*^ MO; Supplementary Fig. 2a–e) generated morphants, which were characterized by abnormal folding of the retinal ventronasal quadrant and the presence of an optic fissure coloboma (lack of fissure fusion; Supplementary Fig. 2f–i; higher doses led to early lethality), often a consequence of abnormal optic stalk development^31^,^41^,^42^. The trunk in morphants, however, appeared normal with no apparent ventral midline defects suggesting that *cdon* knockdown was compatible with axial midline formation. These anterior defects were not found in embryos injected with a control MO (*MO*^*ct*^) nor were rescued by the co-injection of a MO against p53 (*n*=52), which is activated by MO injection and can contribute to off-target effects^43^. Injection of a different *cdon* MO complementary to the 3′ splice site of exon 8 (*Cdon*^*spl8*^ MO) phenocopied *cdon*^*ATG*^ morphants (Supplementary Fig. 3a–b).

**Figure 2:**
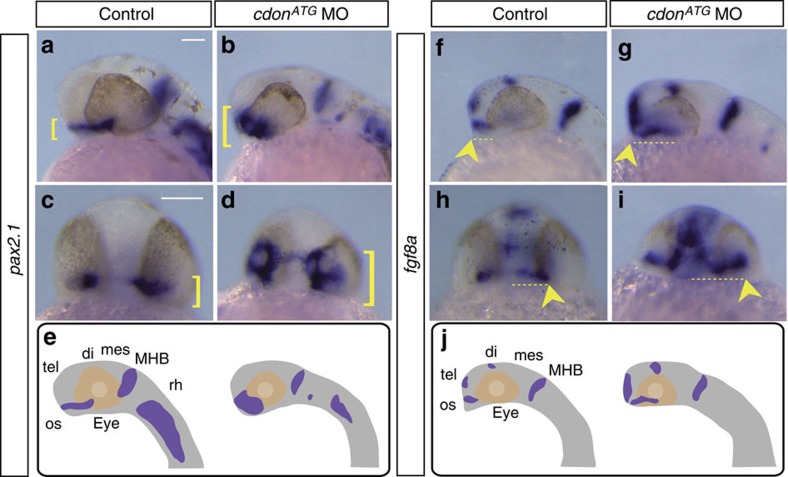
The optic stalk is expanded in *cdon* morphants. (**a**–**j**) *In situ* hybridization analysis for two optic stalk markers, *pax2.1* (**a**–**d**) and *fgf8a* (**f**–**i**) at 28 hpf and 24 hpf, respectively. Embryos are shown in lateral (**a**,**b**,**f**,**g**) and frontal (**c**,**d**,**h**,**i**) views. Expression patterns of both genes are schematically represented in **e** and **j**. In *cdon*^*ATG*^ morphants, *pax2.1* expression is expanded dorsally in the optic stalk (**b**,**d** brackets) when compared with controls (**a**,**c** brackets). *Fgf8a* expression is expanded caudally and laterally in the optic stalk (**g**,**i** arrowhead and dotted lines) as well as in the telencephalon (**g**,**i**) when compared with control embryos (**f**,**h** arrowhead and dotted lines). di, diencephalon; mes, mesencephalon; MHB, midbrain–hindbrain boundary; os, optic stalk; rh, rhombencephalon; tel, telencephalon. Scale bars, 100 μm.

To investigate more precisely the developmental nature of these defects, we analysed the expression pattern of well-established markers of the optic stalk, *pax2.1* and *fgf8a*^32^,^44^. In control embryos, *pax2.1* was expressed in the optic stalk, midbrain–hindbrain boundary (MHB) and along the hindbrain (Fig. 2a,c,e). In most *cdon*^*ATG*^ and *cdon*^*spl8*^ morphants, *pax2.1* was enlarged invading a portion of the ventral optic cup but reduced in the hindbrain (124/140 injected embryos; Fig. 2b,d,e; Supplementary Fig. 3c–d). In lateral views of whole-mounted *cdon*^*ATG*^ morphants, there was an approximate doubling of the visible area of the optic stalks and ventral retina that expressed *pax2.1* (0.007 mm^2^±0.0003 s.e.m; *n*=45) as compared with control embryos (0.0033, mm^2^±0.0002 s.e.m; *n*=28; *T*-test, *P*<0.001). A similar expansion of the optic stalk in *cdon*^*ATG*^ morphants was observed also with an *fgf8a* probe specific (93/112 injected embryos; Fig. 2f–j). *fgf8a* distribution was expanded also in MHB, dorsal diencephalon and telencephalon (Fig. 2f–j). Because coloboma is often associated with ventral retina defects, we also analysed the expression of two ventral retina specific markers, *pdzrn3* (ref. 45) and *rdh10a* (retinol dehydrogenase 10a)^46^. Their distribution was expanded in *cdon*^*ATG*^ morphants in comparison with that of control embryos (Supplementary Fig. 3e–h) but this increase was not associated with an appreciable reduction of the expression of the dorsal retinal marker *tbx5.1* 47 (Supplementary Fig. 3i,j).

Altogether these data support the idea that *cdon* is specifically required for restraining the size of the optic stalk and ventral retina.

### *cdon* counteracts Hh signalling during P-D eye patterning

In mammals, *Cdon* acts as a positive modulator of Hh signalling in the midline and its inactivation in mice causes a microform of HPE^11^,^15^ associate with a decrease in the *Pax2*-positive optic stalk domain^26^, a phenotype opposite to that observed in *cdon* morphants. As a possible interpretation of this discrepancy, we postulated that the mouse optic vesicle phenotype might be a consequence of an early *Cdon* function, which is instead preserved in the knocked-down *cdon*^*ATG*^ morphants, unveiling a later and tissue-specific *cdon* function.

**Figure 3:**
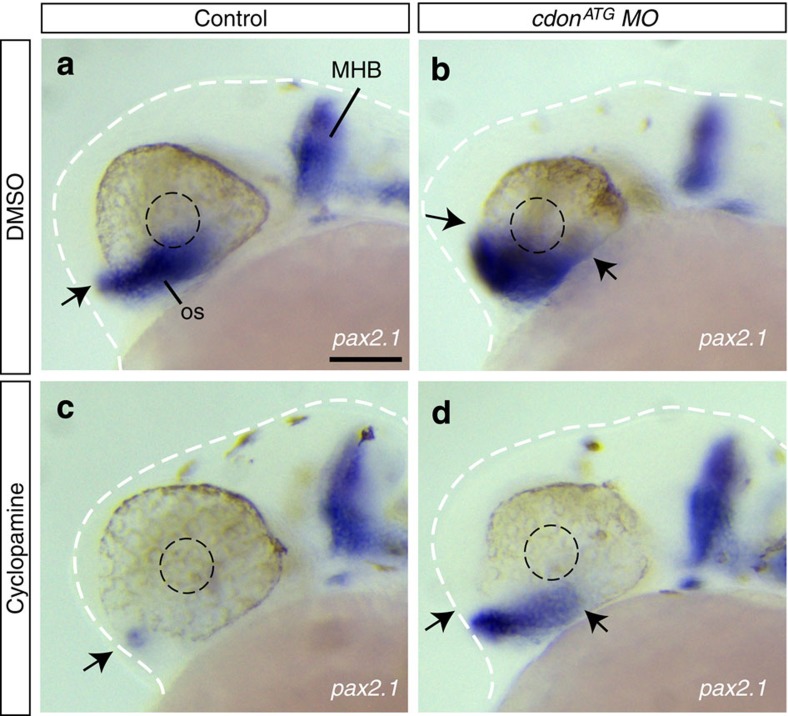
Cdon acts as a negative modulator of Hh signalling. (**a**–**d**) Lateral views of control or *cdon*^*ATG*^ morphants treated with DMSO (vehicle) or cyclopamine from 90% epiboly and analysed with ISH for *pax2.1* expression at 28 hpf. Blocking Hh signalling abolished *pax2.1* expression in the optic stalk in wt embryos (**c**). In *cdon*^*ATG*^ morphants, cyclopamine treatment counteracts the expansion of *pax2.1* overexpression observed in *cdon*^*ATG*^MO injected embryos (**a**,**b**,**d**). The lens and the body are outlined with black and white dashed lines in (**a**–**d**). Arrows indicate the extent of the *pax2.1* expression in the optic stalk. DMSO, dimethyl sulphoxide; MHB, midbrain–hindbrain boundary; oc, optic cup; os, optic stalk. Scale bar, 100 μm.

This function was consistent with Cdon acting as a negative modulator of Hh signalling. If this were the case, inactivation of Hh signalling should rescue the *cdon* loss of function phenotype. To test this hypothesis, we interfered with Hh signalling in *cdon*^*ATG*^ morphants by incubating the embryos in a medium containing the Smo antagonist cyclopamine. As expected, this treatment efficiently abolished the expression of *pax2.1* in the optic stalk of control embryos (Fig. 3a,c). Notably, cyclopamine also counteracted the expansion of *pax2.1* in the optic stalk of *Cdon*^*ATG*^ morphants, generating embryos with an overtly wild-type phenotype (34/35 embryos; Fig. 3a,b,d).

Together these experiments point to the idea that Cdon, when not properly matched by Ptc presence, may act as a negative modulator of Hh signalling in the optic vesicle. This idea was also supported by the expansion of the expression domain of *nkx2.2*, a ventral forebrain Hh target gene^28^, observed in *cdon* morphants as compared with control embryos (Supplementary Fig. 3k–o).

### Cdon function in the eye depends on the Hh binding domain

**Figure 4:**
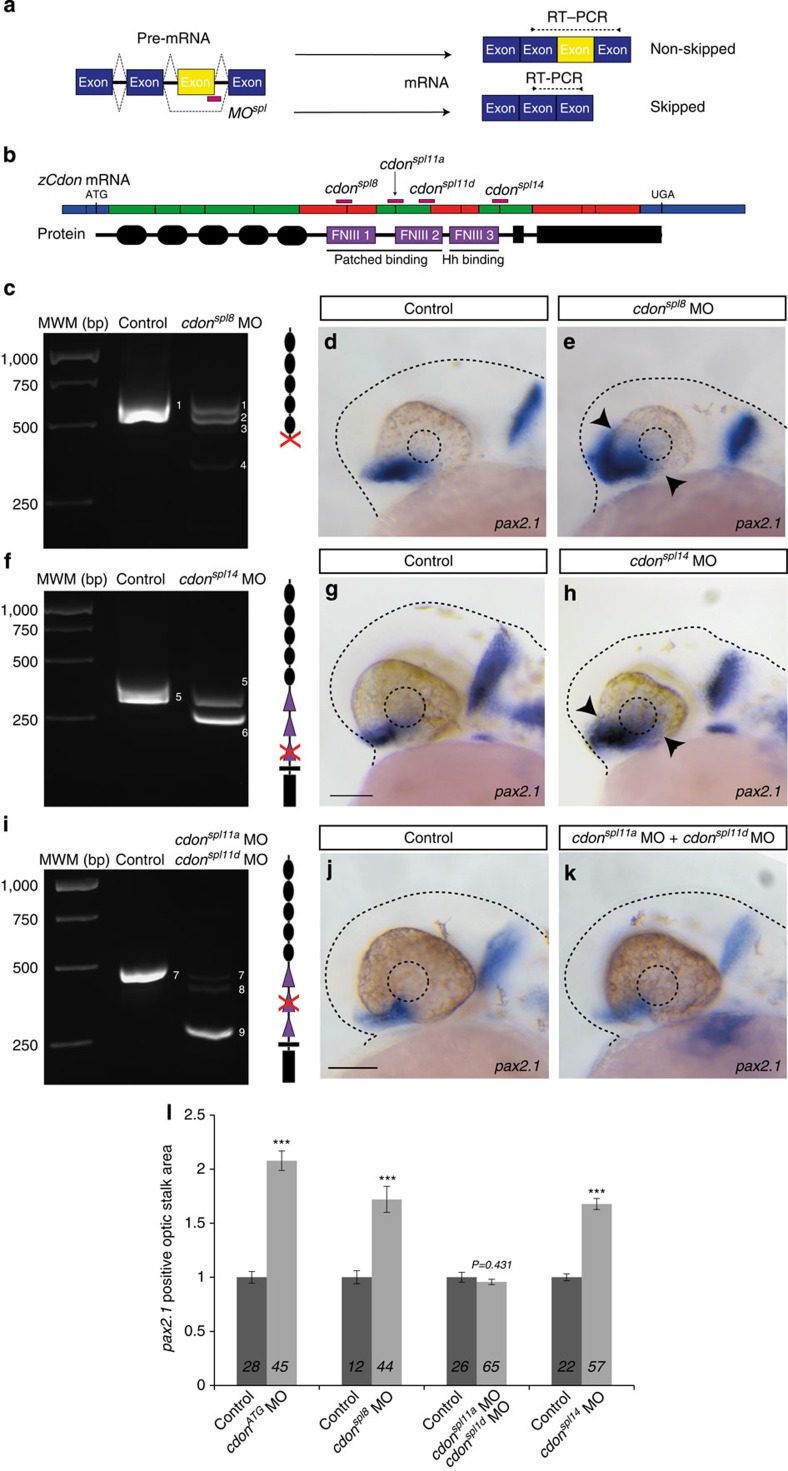
Cdon interaction with Ptc is dispensable for its function in the optic vesicle. (**a**) Schematic diagram of the design of the exon skipping MO used in the study. (**b**) Schema representing the zebrafish *cdon* mRNA (*zcdon*) aligned with the corresponding Cdon protein indicating the domains targeted by *cdon*^*spl8*^, *cdon*^*spl11a*^, *cdon*^*spl11d*^ and *cdon*^*spl14*^ MOs. The exons that encode the 5′ and 3′ UTR regions are depicted in blue; those that, when skipped, generate a translational frame shift are indicated in red; whereas those that, when skipped, maintain the reading frame are indicated in green. (**c**,**f**,**i**) RT–PCR analysis of the exon skipping function of *cdon*^*spl8*^, *cdon*^*spl14*^ and *cdon*^*spl11a*^/*cdon*^*spl11d*^ MOs. For detailed information about the resulting bands, noted 1–9, please refer to Supplementary Fig. 8. (**d**,**e**,**g**,**h**,**j**,**k**) ISH analysis of *pax2.1* expression pattern in *cdon*^*spl8*^ (**d**,**e**), *cdon*^*spl14*^(**g**,**h**) and *cdon*^*spl11a*^/*cdon*^*spl11d*^ (**j**,**k**) MO injected embryos at 26–28 h.p.f. *Pax2.1* expression domain is expanded in *cdon*^*spl8*^ (**d**,**e**) and *cdon*^*spl14*^ morphants (**g**,**h**) in comparison with their respective controls, whereas there was no difference in the *pax2.1* expression domain of control and *cdon*^*spl11a*^/*cdon*^*spl11d*^ morphants (**j**,**k**). (**l**) Quantification of the optic stalk *pax2.1*-positive expression domain in embryos injected with the different MOs at 26–28 h.p.f. (***P< 0.001; Student’s *t*-test). The number of embryos analysed in each case is indicated in each column and are as follows: *Cdon*^*ATG*^ MO injection (control, *n*=28; MO, *n*=45); *Cdon*^*spl8*^ MO injection (control, *n*=12; MO, *n*=44); *Cdon*^*spl11a*^ and *Cdon*^*spl11d*^ MOs injection (control, *n*=26; MO, *n*=65); *Cdon*^*spl14*^ MO injection (control, *n*=22; MO, *n*=57). Error bars represent s.e.m. Scale bars, 100 μm. MWM, molecular weight marker.

Studies in vertebrates have suggested that Cdon interacts with Ptc1 through FnIII(1-2) domains^23^, forming a heteromeric receptor^15^. Cdon further binds Hh through the remaining FnIII(3) domain^16^,^18^. Alignment of the zebrafish, mouse and human CDON sequence revealed conservation of the residues defined as relevant for Ptc and Hh binding^15^,^16^ (Supplementary Figs 4–7). Furthermore, analysis of the zebrafish *cdon* genomic (NC_007129.5) and protein (Q1L8D0) sequences indicated that it was possible to design specific splicing MOs, which would cause skipping of exon 11 or 14 without altering the protein-reading frame (Fig. 4a,b). These MOs, designed as *cdon*^*spl8*^, at the exon-intron or intron-exon boundaries and named *cdon*^*spl14*^, *cdon*^*spl11a*^and *cdon*^*spl11d*^, injected alone (*cdon*^*spl14*^) or in combination (*cdon*^*spl11a*^and *cdon*^*spl11d*^) efficiently generated mutated Cdon versions, considerably reducing the wild-type *cdon* form (Fig. 4). The prevalent mutated forms were, as expected, a frame-shift mutation (*cdon*^*spl8*^) or forms that lacked the functionally relevant sequences of FnIII(3) (*cdon*^*spl14*^) and FnIII(2) (*cdon*^*spl11a*^and *cdon*^*spl11d*^) domains, as determined by PCR with reverse transcription (RT–PCR) analysis of the respective morphants (Fig. 4c,f,i), followed by sequencing of the corresponding bands (Supplementary Fig. 8). Furthermore, transfection of similar constructs into HEK cells demonstrated that the deleted versions of the protein were efficiently inserted into the plasma membrane (Supplementary Fig. 9). We thus expected that *cdon*^*spl11a/spl11d*^ and *cdon*^*spl14*^ MO injections would generate embryos carrying Cdon versions with a reduced capability of interacting with either Ptc1 or Hh.

As observed in *cdon*^*ATG*^MO and *cdon*^*spl8*^ injected embryos (Figs 2a–e and 4c–e,l), *cdon*^*sp14*^ morphants (58/86) were characterized by a significant expansion of the *pax2.1*-positive optic stalk domain, phenocopying the *cdon*^*ATG*^ phenotype (Fig. 4f–h,l). In contrast, *cdon*^*sp11a/ spl11d*^ morphants (110/110) resembled wild-type embryos with no changes in the extent of the *pax2.1* domain despite the evident splicing effect generated by the MOs co-injection (Fig. 4i–k,l).

These results support that, during optic vesicle development, Cdon function depends on Hh binding but likely with a Ptc-independent mechanism. If this were the case, overexpression of Cdon or CdonΔFnIII(2) should rescue the phenotype of *cdon* morphants, whereas CdonΔFnIII(3) should have no effect. To test this hypothesis we took advantage of the tg(*rx3::Gal4*) line^48^. This line enables UAS-mediated overexpression of *cdon* only in the *Rx3*-positive domain (Supplementary Fig. 10a–c), thus offering the possibility of further testing if the zebrafish morphant phenotype is linked to retinal-specific *cdon* function. Injection of UAS::GFP DNA followed by a slightly delayed injection of *cdon*^*ATG*^ MO efficiently drove GFP expression in the developing optic vesicle (Supplementary Fig. 10c) but did not rescue the expansion of the *pax2.1* expression domain caused by *cdon* knockdown (Supplementary Fig. 10f). UAS::Cdon and UAS::CdonΔFNIII(2), but not UAS::CdonΔFNIII(3) DNA, instead significantly counteracted the expansion of the *pax2.1*-positive optic stalk domain characteristic of the morphants (Supplementary Fig. 10d–f).

Together these data support that Cdon expression in the retinal field is required to establish the optic stalk boundary with an Hh dependent but Ptc-binding independent mechanism.

### Retinal *Cdon* expression preserves the retina/stalk boundary

In *Drosophila* both Ihog and Boi can sequester the amount of Hh available thereby limiting long-range signalling^19^,^36^,^37^,^38^. We thus hypothesized that Cdon expressed in the retinal primordium could bind Hh preventing its dispersion into the future retinal domain. In this case, loss of *Cdon* function should expose retinal cells to Hh, thereby expanding the stalk domain.

**Figure 5:**
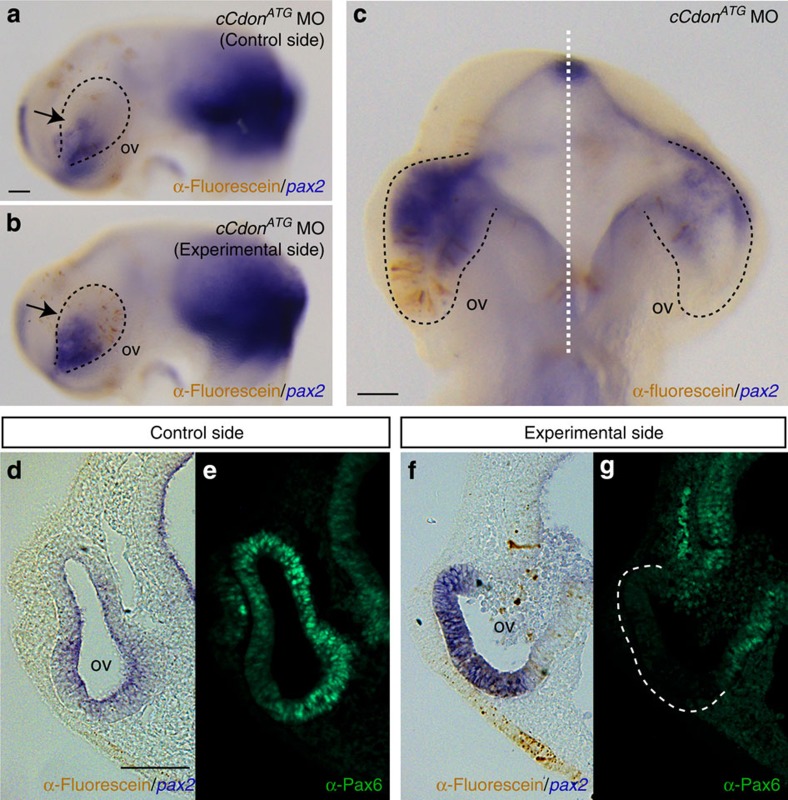
Localized interference with *Cdon* expression in the optic vesicles expands the distal optic stalk domain. (**a**–**f**) Lateral (**a**,**b**) and ventral (**c**) views of the control (**a**) and experimental (**b**) optic vesicles (ov) of HH14 chick embryos with unilateral focal electroporation of a carboxyfluorescein conjugated *cCdon*^*ATG*^ MO at HH8. Embryos were hybridized for *pax2* (blue signal) and immunostained with anti-fluorescein antibodies (brown signal) to detect MO distribution (**a**–**d**,**f**). Pax6 immunohistochemistry was performed in cryostat sections of electroporated embryos (**e**,**g**). *Pax2* expression is expanded to the entire optic vesicle of *cCdon*^*ATG*^ MO-treated embryos (**b**,**c**) in comparison with the non-electroporated control eye (**a**,**c**). *Pax2* expansion is associated with a reduction of Pax6 distribution in the retina (**f**,**g**) in contrast to a wild-type condition (**d**,**e**). The optic vesicles are outlined with black (**a**–**c**) or white (**g**) dashed lines. Scale bar, 50 μm.

To test this possibility, we interfered with *Cdon* expression in a spatiotemporal controlled manner, taking advantage of the evolutionary conserved distribution of *Cdon* in the chick optic vesicles (Supplementary Fig. 1o,s). A c*Cdon*^*ATG*^*-*tagged^*-*^MO was forced by focal *in ovo* electroporation into the eye anlage of HH8 embryos, when *Cdon* expression in vesicle appears (Supplementary Fig. 11). Targeted embryos showed an expansion of *pax2* expression, which was not observed in embryos electroporated with an equally tagged *cMO*^*ct*^ or in the contralateral eye of unilaterally c*Cdon*^*ATG*^ electroporated embryos (Fig. 5a–d,f). This phenotype resembled that of gain of midline-derived Hh signalling^32^,^49^. Expansion of *pax2* expression was associated with a clear reduction of Pax6 distribution in the optic cup (Fig. 5e,g), well in agreement with the notion that Pax2 and Pax6 regulate each other’s activity^29^.

Together these data indicate that *Cdon* expression in the retinal primordium controls the balance between Pax2/Pax6 thereby establishing the boundary between the optic stalk and the optic cup. Considering that *Pax2* is an Hh target^30^,^32^,^33^, the position of this border depends upon Hh protein distribution, which, in turn, may be influenced by Cdon acting as a barrier.

### Cdon controls Hh dispersion in the neuroepithelium

**Figure 6:**
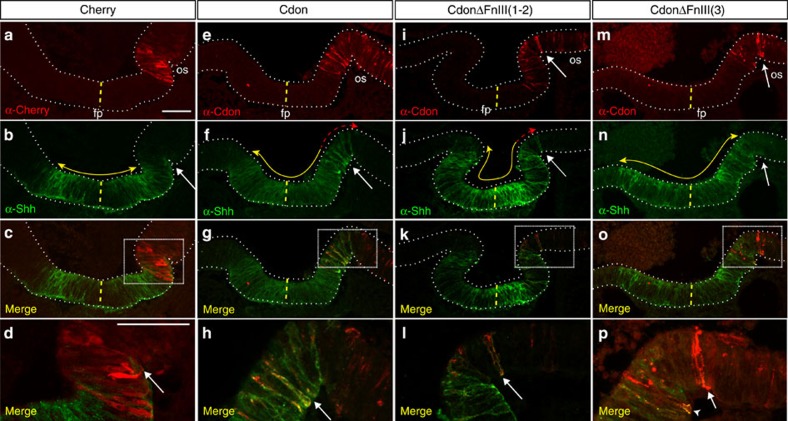
*Cdon* overexpression modifies Shh protein distribution in the optic stalk neuroepithelium. (**a**–**p**) Confocal analysis of coronal sections at the level of the optic vesicle of HH14 chick embryos electroporated at HH8 with a construct carrying mCherry (**a**–**d**), Cdon (**e**–**h**) or its deleted derivatives lacking the Ptc (CdonΔFnIII(1-2)) (**i**–**l**) or Shh (CdonΔFnIII(3)) (**m**–**p**) binding domains. Sections were immunostained with antibodies against mCherry (**a**), Cdon (**e**,**i**,**m**) and Shh (**b**,**f**,**j**,**n**). Note the localization of Shh protein in Cdon-positive cells—particularly in their basal regions (**f**,**h** white arrow)—falling outside of the Shh-expression domain (**e**–**h**). A similar accumulation is observed in the presence of the CdonΔFnIII(1-2) construct (**i**–**l** white arrow) but was hardly detectable in mCherry or CdonΔFnIII(3) expressing cells falling outside of the Shh domain (**a**–**d** and **m**–**p**). The midline is indicated with a yellow dotted line. The normal extent of Shh distribution is indicated with a yellow line, whereas extended Shh localization is indicated in red. In **p** the arrowhead points to a Cdon-positive cell within the Shh domain, whereas the arrow points to the first Cdon-positive cell falling outside the Shh domain. fp, floor plate; os, optic stalk. Scale bar, 50 μm.

We next investigated if Cdon expression modifies the distribution of the endogenous Hh protein from the ventral hypothalamus. To this end, we used *in ovo* electroporation and expressed mouse *Cdon* and its deleted derivatives^17^ ectopically into the optic stalk of HH8 chick embryos, close to the Hh producing ventral midline. Successfully targeted embryos were then co-immunostained with antibodies against Cdon and Shh. In embryos electroporated with a control membrane Cherry-expressing vector (Fig. 6a–d) or in the non-electroporated half of the embryo (Fig. 6e–h), Hh signal was localized in the ventral neuroepithelium with a symmetric distribution that covered about 125 μm from the midline. This symmetric distribution was no longer observed when Cdon was expressed in the adjacent optic stalk neuroepithelium (25/25 of positive embryos). In this case, the ectopic Cdon-positive cells closest to the Hh source presented an accumulation of Hh immunoreactivity, particularly evident at the basolateral side of the cells. This strong signal was mostly limited to the Cdon-positive cells proximal to the Shh domain and less evident in Cdon-positive cells that occupied a position more distal to the Shh source (Fig. 6e–h; Supplementary Fig. 12d–f,m,n). Notably, the effect of Cdon on Hh protein distribution was independent of Cdon interaction with Ptc1 because ectopic expression of CdonΔFnIII(1-2) caused a similar predominant accumulation of Hh at the most proximal Cdon-positive cells (Fig. 6i–l; Supplementary Fig. 12g–i,m,n). On the contrary, forced expression of CdonΔFnIII(3) had little effect on the symmetric Hh distribution (Fig. 6m–p; Supplementary Fig. 12j–n), although proximal cells expressing this Cdon mutated version appeared to bind more ligand than control cells, opening the possibility that Cdon residues other than those present in the FNIII(3) domain partially contribute to Shh binding. Alternatively, this Cdon deleted form may enhance the Hh binding efficiency of other receptors expressed in the neuroepithelium.

**Figure 7:**
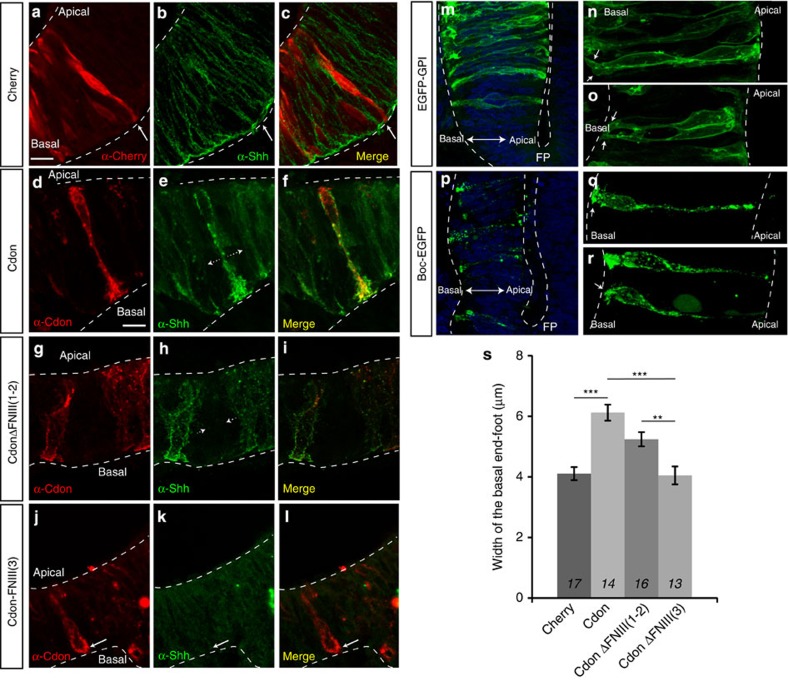
Cdon and Boc promote morphological changes of the neuroepithelial basal side where Shh preferentially accumulates. (**a**–**r**) Confocal analysis of coronal sections at the level of HH14 chicken optic stalk (**a**–**l**) and HH10 neural tube (**m**–**r**) electroporated at HH8 with a construct carrying mCherry (**a**–**c**) Cdon (**d**–**f**), CdonΔFnIII(1-2) (**g**–**i**), CdonΔFnIII(3) (**j**–**l**), EGFP-GPI (**m**–**o**) or Boc-EGFP (**p**–**r**). Sections in (**a**–**l**) were immunostained with antibodies against Cherry (**a**) Cdon (**d**,**g**,**j**) and Shh (**b**,**e**,**h**,**k**). Images in (**n**,**o**) and (**q**,**r**) are high magnification views of cells shown in (**m**,**p**) respectively. Note how cells electroporated with Boc-EGFP (**q**,**r**, arrows), Cdon (**d**) or CdonΔFnIII(1-2) (**g**) present an enlarged basal end-foot when compared with EGFP, mCherry or CdonΔFnIII(3) neuroepithelial cells (**n**,**o** and **a**,**j** arrows). This enlarged end-foot is a preferential site of Shh accumulation (**e**,**f**,**h**,**i**). Note also the absence of Shh signal in the cells immediately surrounding the Cdon-positive cells (**e**,**h**, dotted line arrows). This distribution is not observed n cells expressing mCherry (**b**,**c**) or CdonΔFnIII(3) (**k**,**l**). (**s**) Quantification of the basal end-foot width of neuroepithelial cells electroporated with Cdon and its deleted versions. The number of cases analysed for each data set is indicated in the respective column. The numbers of quantified cells are indicated in the graph labels and are as follows: Cherry, *n*=17; Cdon, *n*=14; CdonΔFnIII(1-2), *n*=16 and CdonΔFnIII(3), *n*=13. Error bars represent s.e.m. (**P<0.01, ***P<0.001; Student’s *t*-test). There is no statistical difference in the basal end-foot width between Cdon and CdonΔFnIII(1-2) expressing cells (*P*=0.066, Student’s *t*-test) or between Cherry and CdonΔFnIII(3) expressing cells (*P*=0.998; Student’s *t*-test). Scale bar, 10 μm.

Notably, Cdon-positive neuroepithelial cells presented a significantly enlarged basal end-foot as compared with Cherry electroporated control cells (Fig. 7a–f,s; Supplementary Fig. 13a–f). Furthermore, in control cells the end-foot was rather simple, whereas in Cdon-positive cells the end-foot was decorated by several filopodial-like extensions that appeared to be a main site of Hh accumulation (Fig. 7d–f; Supplementary Fig. 13d–f). Consistent with the idea that Cdon acts as a ‘sink’ for Hh protein, cells immediately surrounding a Cdon-positive cell were devoid of Hh immunoreactivity (Fig. 7e,f; Supplementary Fig. 13e,f). A similar significant enlargement of the basal end-foot associated with the presence of some cytoplasmic extensions was observed also in cells that ectopically expressed CdonΔFnIII(1-2) (Fig. 7g–i,s; Supplementary Fig. 13g–i), but not in those carrying the CdonΔFnIII(3) protein (Fig. 7j–l,s; Supplementary Fig. 13j–l). Together these results indicate that Cdon influence of basal end-foot morphology is independent from Cdon interaction with Ptc but is likely linked to Hh interaction.

Similar results were observed when the related Boc-EGFP was ectopically expressed in the posterior neural tube supporting the idea that the two proteins can act in a similar manner^23^,^24^. Boc-positive neuroepithelial cells accumulated Shh (not shown) and presented an enlarged basal end-foot decorated by several filopodial-like extensions (Fig. 7m–r). This end-foot was larger (6.2 μm ±0.7 s.e.m; *n*=7; *P*≤0.001) than that of GFP-positive neuroepithelial cells (2.6 μm±0.3 s.e.m.; *n*=6).

All in all, these results support the idea that Cdon and Boc localize predominantly at the basal surface of the neuroepithelial cells enriched in filopodial-like extensions. These protrusions may serve to enhance Hh binding in Cdon/Boc expressing cells, thus modifying Hh distribution and thereby its activity.

## Discussion

Our study provides evidence that Cdon-mediated interference with Hh ligand dispersion is a mechanism by which Hh signalling information can be regulated in vertebrates. We show that Cdon expression in the retinal neuroepithelium serves to prevent the activation of the Hh target gene *pax2*, thereby controlling the proximal-distal patterning of the optic vesicle. We propose that mechanistically this occurs because, in the absence of Ptc co-expression, Cdon acts as a sink for Hh proteins that predominantly accumulate at the filopodial-enriched basolateral side of Cdon-expressing neuroepithelial cells. A similar sink mechanism, mediated by Boi or Ihog, seems to limit Hh long-range distribution in *Drosophila*^36^,^37^,^38^. Therefore we propose that, in the absence of Ptc interaction, Cdon/Boi/Ihog—and perhaps Boc^28^—have an evolutionary conserved function as Hh decoy receptors. This mechanism of limiting Hh activity acts in parallel to other, more intensively studied mechanisms, for example, negative feedback regulation. A case in point is Hh-mediated control of the expression of the Ptc receptor, which, in turn, inhibits pathway activation^50^.

Our notion of Cdon-mediated regulation of Hh signalling is supported by the analysis of Cdon effect on Hh protein distribution and by the consequences of graded and spatiotemporal controlled interference with *cdon* expression in both zebrafish and chick-developing optic vesicle. The eye phenotype resulting from this interference was compatible with that observed after gain of Hh signalling function in the optic vesicle, which is characterized by the expansion of the *pax2*-positive optic stalk domain^30^,^31^,^32^,^51^, indicating that Pax2 is an Hh target. Consistent with these observations, *cdon* knockdown caused an expansion of *pax2.1* eye expression, which was rescued by interfering with Hh signalling activation and by the localized overexpression of *Cdon* within the eye field. Furthermore, ventral retina defects similar to those of *cdon* morphants have been reported in other zebrafish lines, in which Hh signalling is overactivated, for example, as in the *blowout*^34^, *uta1*^52^ and *aussicht* lines^53^, in *Zic2a* morphants^49^ or after Hh overexpression in zebrafish^30^,^32^ and chick embryos^51^.

Notably, our findings are also in good agreement with studies in the teleost fish *Astyanax mexicanus,* a natural example of the consequence that Hh signalling expansion has on eye development. In their natural environment, the *Astyanax mexicanus* exists in two forms: a surface-dwelling river morph and a cave-living blind morph (cavefish). In the embryonic cavefish, the ventral expression domains of *Hh* and *tiggy-winkle (twhh),* a family member that shares Hh functions in the developing eye^30^, are expanded in the forebrain^54^,^55^, Hh target genes, including *pax2,* are hyperactivated and the ventral quadrant of the retina fails to develop^55^. These defects coincide with those of the *cdon* morphants, tempting us to anticipate that in the cavefish *cdon* expression might be altered.

Despite the clear rescue of *cdon* morphant phenotype by inhibition of Hh signalling, we could not detect an evident expansion or upregulation of the expression of Hh target genes (*ptc1 gli2*, *gli2b*, *gli3*, *boc* and *nkx2.1*) in *cdon* morphants, with the exception of *nkx2.2*, which is broadly expressed in the anterior hypothalamic region. Invariance of target gene expression after gain of Hh function has been reported in other studies, in which the phenotype could be nonetheless rescued by modifying the activity of other Hh pathway components^49^,^53^. These observations may reflect differential sensitivity of target genes to Hh concentrations^56^. *Ptc*, *gli2*, *gli2b*, *gli3* and *nkx2.1* likely respond to high midline-derived Hh levels, whereas *nkx2.2*, given its broad expression, might be more sensitive to local Hh modifications caused by interference with *Cdon* expression.

*Cdon* has a transient but conserved expression in the prechordal plate mesendoderm, notochord and ventral neural tube midline, which all produce Hh proteins. Recent studies have shown that Ihog regulates the release of Hh from the producing cells with high levels of Ihog blocking Hh release or transport^4^,^36^. At the same time, Ihog favours the formation of cytonemes that ensure long-range transport of Hh molecules^6^ and in the receiving cells increases Hh signalling^19^. In a similar manner, Cdon may control Hh dispersion shaping the gradient responsible for the specification of ventral fates, including among them the optic stalk. Thus, complete loss of Cdon function would overall reduce Hh dispersion leading to a HPE phenotype, as observed in humans carrying CDON mutant alleles^15^ or in *Cdon* null mice^11^,^20^,^25^,^57^. *Boc*, *Gas1* or *LRP2* might reinforce this Cdon function in vertebrates, as suggested by the stronger phenotype observed in compound *Cdon*^−/−^*;Boc*^−/−^ mouse embryos^24^. Although we cannot completely exclude that this compensation might explain the absence of midline defects in our *Cdon* morphants, we believe that our downregulation of *Cdon* function in both zebrafish and chick bypasses this early function, which is likely responsible for the reduced expression of *Pax2* observed in the optic vesicle of *Cdon*^−/−^ mouse embryos^26^. The incomplete downregulation of *cdon* in zebrafish has, however, the advantage of unveiling a later *Cdon* effect: protecting the retinal neuroepithelium from Hh signalling. In absence of Ptc, Cdon-mediated tight Hh binding would not lead to signalling activation but rather to precluding Hh dispersion. Therefore, the same Ihog/Cdon function of tightly binding the available Hh may limit long-range or enhance signalling depending on their expression, their distance to the Hh source or the concomitant presence of the Ptc receptor.

This Ptc1-independent decoy function of Cdon is supported by the complementary expression pattern of Cdon and Ptc in the eye and other neural regions. Furthermore, the use of exon skipping MO demonstrated that CdonΔFnIII(3)—deficient in Hh binding—mimicked *cdon*^*ATG*^ morphant phenotype, whereas removal of *cdon* Ptc-binding domain had no obvious effect on eye development. Analysis of the effects of Cdon and its FNIII(2) and FNIII(3) deleted derivatives in localized rescue experiments and on the endogenous Hh distribution led to similar conclusions. We show that neuroepithelial cells expressing Cdon accumulate Hh protein on their surface independently from the presence of the Ptc interacting domain, indicating that this Cdon variant still effectively binds Hh, well in agreement with studies with human CDON mutated alleles^15^ and with Ihog mutant forms in *Drosophila*^19^. Cdon ectopic expression did not change the fate of targeted cells into Hh-producing cells, as determined by ISH analysis (not shown). Thus, we propose that Cdon as well as Boc can tightly bind Hh, depleting the amount of morphogen present in the surrounding cells, as observed in our studies (Fig. 7). This strong accumulation prevents further diffusion of the ligand, thereby blocking pathway activation.

What is the destiny of the accumulated Hh protein is just a matter of speculation at the moment. In a simple view the protein could be endocytosed and degraded with a mechanism similar to that described for Glypican3 (ref. 58), Ptc^34^,^59^,^60^ or the Hh interacting protein^61^. An alternative possibility comes from the observation that Cdon and Boc expression is associated with the presence of filopodial-like extensions at the basal side of neuroepithelial cells. Although shorter, these extensions may be related to the cytonemes initially described in *Drosophila* as structures implicated in morphogen transport^62^ and recently proven to be at the base of Hh long-range distribution in *Drosophila*^6^ and in the vertebrate limb bud^8^. Cytonemes are present in both Hh producing and receiving cells and are enriched in Ihog^6^ or Cdon^8^. It is possible that in cells that should not receive Hh signalling, as the presumptive retinal epithelium, Cdon-enriched extensions could serve to reshuffle Hh to the nearby Hh responding optic stalk cells. In this way, the activity of overly dispersed Hh will be maximized by being reconvened at the responding cells farthest away from the Hh source, thus reinforcing signalling activation in these cells. Further studies are needed to establish if these extensions are specific features of Cdon/Boc expressing cells or are normally present in all neuroepithelial cells in labile forms undetectable with conventional fluorescent techniques, which become stabilized by the expression of these proteins, as suggested in *Drosophila*^6^. Notably, basolateral protrusions have been very recently described in the mouse neural plate^63^.

In *Drosophila*, Boi and Ihog have a redundant function in Hh pathway activation^19^ and can sequester and titrate the amount of ligand available for Ptc binding, thus limiting long-range signalling^19^,^37^,^38^,^64^. Ihog is also involved in cytoneme-based Hh signalling^6^. Thus, at least Ihog seems to have both a positive and negative effect on Hh signalling activation^38^. Our study, together with the observations that Cdon, Boc and Gas1 have redundant functions as Hh co-receptors^17^,^20^,^22^,^24^,^57^, indicates that this dual activity is conserved in the vertebrate Cdon homologue and perhaps also in Boc. Indeed, Boc overexpression correlates with the presence of filopodial-like extensions at the basolateral side of the neuroepithelium (Fig. 7) and its expression seems to protect mouse ipsilateral projecting retinal ganglion cells from Shh activity^65^. Similarly, high levels of Boc expressed in the zebrafish jaw have been proposed to bind Shh and limit the ability of Hh to activate chondrogenesis and proliferation in this tissue^28^. Whether Cdon and Boc participate in Hh release/transport, as Ihog does, remains to be determined. Furthermore, it might be worth exploring whether Cdon has Hh unrelated function in the developing eye. Indeed independent of its Hh-related activity, Cdon has been shown to interact with N-cadherin^66^ which, in turn, is relevant to proper optic fissure closure^67^.

## Methods

### Maintenance of fish lines and chicken embryos

Adult zebrafish (*Danio rerio*) and the Tg(*rx3:Gal4-VP16*) line^48^ were maintained at 28.5 °C on a 14/10 h light/dark cycle. Embryos (AB/Tu or WIK strains) were raised at 28 °C and staged according to hours post fertilization (h.p.f.) and morphology^68^. Embryos were growth in E3 medium (NaCl, 5 mM; KCl, 0.17 mM; CaCl_2_, 0.33 mM; MgSO_4_, 0.33 mM, 5.10% Methylene Blue). We used chicken embryos (*Gallus gallus domestica*) of the White Leghorn breed raised in the Santa Isabel farm (Córdoba, Spain). The procedures used in the study were approved by the Ethical Committee for Animal Experimentation of the Consejo Superior de Investigaciones Cientificas (CSIC).

### Embryo injections

The MOs used in this study are listed in Supplementary Table 1. The *MO*^*ct*^ and *cMO*^*ct*^ are standard control MOs from Gene Tools. MOs were injected at concentrations listed in Supplementary Table 1 (1nl) by using a microinjector (Femtojet/ Eppendorf or IM-300/Narishige) in zebrafish embryos at one-cell stage. To determine *cdon*^*ATG*^ MO efficiency, the *cdon*^*ATG*^ MO or *MO*^*ct*^ were co-injected into medaka fish embryos at one-cell stage with the RNA coding GFP alone or with the cRNA (50 ng μl^−1^) of a reporter construct (5′*cdon*-GFP) carrying the *cdon* 5′ sequence upstream of the GFP sequence and the knockdown of GFP expression was analysed. The exon removal efficiency of *cdon*^*spl8*^, *cdon*^spl11a^, *cdon*^spl11d^ and *cdon*^spl14^ MO was verified by RT–PCR using the cDNA of the respective morphant embryos at 24 h.p.f. The primers used to test effective exon skipping are the following: *cdon*^*spl8*^MO, fwd: 5′-TATCCATCCTGCGAGGTTTG-3′, rev 5′-GACCTCATACAGGCTGGACG-3′; *cdon*^spl11a^ and *cdon*^spl11d^ MOs fwd: 5′-CCAATAAGAATCCCTCCA AAG-3′, rev 5′-CCAGCGTAACAGAATCTGAG-3′; *cdon*^spl14^ MO fwd: 5′-ACACTCCATCCAGCAATAAC-3′, rev 5′-TCCTCCATAAGCACATGACG-3′. PCR products were run in agarose gels (1–2%) and visualized with FastRed (Biotium). The different bands were excised from the gel, ligated by T-cloning in the pSC-A vector (Stratagene) and sequenced. Sequences are reported in Supplementary Fig. 8. *cdon*^*spl8*^ MO injection generated three aberrant *cdon* mRNAs: band 2 carrying a deletion of 73 bp that causes a frame shift; band 3 carrying a 162 bp deletion and band 4 corresponding to the frame-shift deletion of exon 8 (Fig. 4c). *cdon*^*spl14*^ MO injection generated one aberrant *cdon* mRNAs: band 6 corresponding to the exon14 skipping (Fig. 4f). *cdon*^*sp11a*^ and *cdon*^*spl11d*^MOs co-injection generated two aberrant *cdon* mRNAs: band 8 carrying a deletion of 29 bp that causes a frame shift and band 9 corresponding to the exon 11 skipping (Fig. 4i). The p53 MO was used according to Robu *et al.*^43^

### Tissue processing and immunochemistry

Fish, chicken and mouse embryos were fixed by immersion in 4% paraformaldehyde-phosphate buffer (wt/vol) overnight at 4 °C. Embryos were then washed in phosphate buffer saline (PBS), incubated in a 15% sucrose-PBS solution (wt/vol), embedded and frozen in a 7.5% gelatin in 15% sucrose solution (wt/vol). Cryostat sections were processed for IHC or for *in situ* hybridization (ISH). To detect endogenous Shh distribution, chick embryos were instead fixed in ethanol 95%/acetic acid 1% for 20 min. Cryostat sections or whole embryos were stained by a standard protocol using antibodies against the following antigens: Cdon (1:100, R&D), Pax6 (1:500, Covance), Pax2 (1:500, Zymed), Fluorescein (1:500, Roche), Shh (1:500, 5E1 Hybridoma bank), HA (1:250, Sigma). For Hh and Pax2 immunostaining in zebrafish, we adapted the protocol for tissue slices^69^ by boiling the embryos directly in citrate buffer 10 mM at 110 °C during 5 min. Embryos were thereafter washed in PBS and incubated for two days in the following primary antibodies: anti-Shh (1:50, R&D) and anti-Pax2 (1:50, Zymed). Incubation with appropriate secondary antibodies was performed with standard procedures.

### *In situ* hybridization

Chicken embryos were hybridized using digoxigenin- or fluorescein-UTP-labelled antisense riboprobes for *pax2* and *cdon*. Probes were visualized with NBT/BCIP (dark blue). Zebrafish embryos were hybridized *in toto* by standard procedures using the following digoxigenin-UTP-labelled antisense riboprobes: *tbx5.1*, *fgf8*, *pax2.1*, *ptc1*, *shh, nkx2.1, nkx2.2* and *rdlh10a*. Chicken embryos were hybridized *in toto* by standard procedures using the following digoxigenin-UTP-labelled antisense riboprobes: *pax2* and *cdon*. The zebrafish *boc* and *cdon* coding sequences were obtained by RT–PCR from cDNA of embryos collected at different developmental stages using the following primers (*boc:* fwd: 5′-CATCGATCCTTTCAATGCAAG-3′, rev: 5′-GGGAGTATTCTTGTTTCATCCA-3′; *cdon:* fwd: 5′-CATCGGGAGAATGTGTTTCG-3′, rev: 5′-TCCACCAATATCTTCATTCG-3′) and cloned in pDrive vector (Qiagen).

### mRNA and cDNA synthesis and cloning

Embryos were injected with different splicing MOs at one-cell stage and at 24 h.p.f. were frozen in dry ice. RNA purification was performed with PureLink miniKit (Invitrogen) and cDNA was synthesized with iScript cDNA synthesis Kit (Bio-Rad). 5′*cdon*-GFP construct was obtained by fusing the RT–PCR amplified *cdon* coding sequence upstream of the *GFP* sequence in a pCS2 vector (pCS2-5′*cdon*-*GFP*). Capped RNA (cRNA) was synthesized using pCS2-5′*cdon*-GFP linearized with NotI as a template and the mMessage mMachine SP6 Kit (Ambion). The resulting mRNA was purified using RNeasy Mini Kit (Qiagen). 5′*cdon*-GFP mRNA was injected (1nl) at 80 ng ul^−1^. To generate the UAS constructs, mCdon-HA was amplified from pCIG-mCdon using the Expand High Fidelity PCR system (Roche) and the following primers fwd: 5′-ATAGAATTCACGTGCTGGTTATTGTGCTG-3′, rev: 5′-ATAGAATTCAGCGGCTTCGGCCAGTAACG-3′. The PCR product was cloned with T-cloning in pSC-A (Stratagene). The vector was digested with EcoRI/FbaI and the band corresponding to mCdon-HA was cloned into the pCS2 vector digested with EcoRI. The pCS2-mCdon vector was verified by restriction analysis with EcoRV/XhoI digestion and by DNA sequencing. Versions of Cdon lacking FnIII-2 or the FnIII-3 were generated by PCR amplification with a PfuUltra DNA polymerase (Agilent) from the pCS2-mCdon vector using the following 5′phosphorilated primers 5′-CAGGTGGCCGGCTTCCCAAATC-3′, 5′-ATGCCTGGAGGAATCCGTAAG-3′ for deletion of FNIII(2) and 5′-GGAGCTTCCGACTATCCCGTG-3′, 5′-CACCTGGTAAGGACGGGATGC-3′ for deletion of FNIII(3). The amplified products were ligated (Ligase, Roche) overnight at 16 °C, digested with DpnI enzyme to remove the non-amplified DNA and then transformed. The pCS2-mCdon, containing an HA tag, and its derivatives (pCS2-CdonΔFnIII(2) and pCS2-CdonΔFnIII(3)) were transfected in HEK cells and the expected products were verified by immunostaining and western blot analysis with anti-Cdon and anti-HA antibodies. pCS2-mCdon, pCS2-CdonΔFnIII(2) and pCS2-CdonΔFnIII(3) vectors were digested with ClaI and the product was treated with Klenow polymerase for blunting DNA ends. The product was digested with NotI/SalI and run in an agarose gel. The band of approximately 4 Kb was excised from the gel, purified and ligated in pBR-Tol2-UAS:MCS-UAS:GFP previously digested with EcoRV/NotI (in short, UAS::GFP, which was kindly provided by Dr Masa Tada). The obtained vectors UAS::Cdon, UAS::CdonΔFnIII(2) and UAS::CdonΔFnIII(3) were verified by restriction analysis with KpnI and EcoRV/BglII digestion, PCR amplification and sequencing. Efficient expression and membrane localization of the constructs were verified by DNA microinjection in the *rx3::Gal4* transgenic line and α-HA immunostaining at 20 h.p.f. (Supplementary Fig. 10).

### Deletion constructs of Cdon and Boc

pCIG-mCdon-HA (pCIG-mCdon), pCIG-rCdon-FnIIIΔ12-HA (CdonΔFnIII(1-2)) and pCIG-mCdon-FnIIIΔ3-HA (CdonΔFnIII(3)) vectors were kindly provided by Dr B. Allen^17^. pEGFP-mBoc expression vector (Boc-EGFP) was kindly provided by Dr Ami Okada^65^. Versions of Boc lacking FnIII-2 or the FnIII-3 were generated by PCR amplification with a PfuUltra DNA polymerase (Agilent) from the pEGFP-mBoc plasmid using the following primers: Boc-GFPΔFnIII-2 fwd: 5′-GTGTCAGGCTACAGTGGC-3′, rev: 5′-ATGGTCAGGCTGGCTGCT-3′; Boc-GFPΔFnIII-3 fwd: 5′-TTTTCTGGTCAGCCTGGA-3′, rev: 5′-CCTCTCATATACACGGCC-3′. The amplified products were ligated (Ligase, Roche) overnight at 16 °C, digested with DpnI enzyme to remove the non-amplified DNA and then transformed.

### Chicken electroporation

Fertilized chicken eggs were incubated at 38 °C to reach the desired stage. A carboxyfluorescein-tagged^70^ chicken-specific MO (c*Cdon*^*ATG*^MO) was designed to be complementary to the translation start site sequence of Cdon (NCBI Reference Sequence: XM_417853.4). The MO efficiently reduced the levels of the endogenous protein (Supplementary Fig. 11). The selected vectors (1.5 μg μl^−1^), *cMO*^*ct*^ or *cCdon*^*ATG*^ were injected *in ovo* in the eye primordium of HH8 chick embryos right at the appearance of *cdon* expression in the forming vesicle. The electrodes were placed at the head sides. Focal electrical discharges were applied with the following conditions: voltage, 14V; pulses, 5; interval, 300 ms and pulse length, 50 ms. Thereafter, eggs were sealed and incubated at 38 °C until embryos reached HH14, when *cdon* mRNA localizes to the entire retina (Supplementary Fig. 11). Embryos were then selected according to GFP expression and fixed for further processing.

### Rescue experiments

*Cdon*^*ATG*^*MO* and UAS::GFP, UAS::Cdon, UAS::CdonΔFnIII(2), and UAS::CdonΔFnIII(3) DNAs were co-injected in the transgenic line Tg(rx3:Gal4-VP16). DNA (35 pg) was injected into the cell of one-cell stage embryos, whereas *Cdon*^*ATG*^*MO* (1nl of at 160 uM) was introduced in a second round of injections into the yolk at the two–four-cells stage. Embryos were let to develop for 26–28 h in E3 medium at 28 °C. Embryos showing a homogeneous expression of GFP in the retina were selected and further processed for *pax2.1* ISH.

### Cell transfection

Sub-confluent Human Embryonic Kidney 293T (HEK293) cells were transiently co-transfected with the pCIG-Cdon-HA and derivatives or with the pEGFP-mBoc or derivative-plasmids using the FuGENE HD Transfection Reagent (Roche). After 48 h, cells were visualized by fluorescence in a confocal microscope or scraped in lysis buffer for western blot analysis.

### Western blotting

HEK293 cell or tissue samples (electroporated chicken eyes) were collected or dissected, treated with lysis buffer (150 mM NaCl, 1% NP40, 50 mM Tris pH8 ) and denatured in protein loading buffer (50 mM Tris–HCl pH 6.8, 2% SDS, 10% glycerol, 1% β-mercaptoethanol, 12.5 mM EDTA and 0.02% bromophenol blue). Proteins were resolved by 6% SDSP-gels, blotted onto a PVDF membrane, blocked in 5% milk in PBS Tween-20 for 1 h, incubated with Cdon polyclonal antibody (R&D system) or HA polyclonal antibody (Sigma) in blocking buffer overnight at 4 °C, washed four times with PBST, and incubated for 1 h with peroxidase-conjugated secondary antibody. Labelled proteins were detected with the chemiluminescence reagent ECL (Amersham Biosciences).

### Treatment with Hh signalling inhibitor

Cyclopamine (Calbiochem) was used to antagonize Hh signalling. Embryos at 90% epiboly were incubated in E3 medium containing 100 μM Cya dissolved in dimethyl sulphoxide at 28 °C. Embryos were fixed in 4% paraformaldehyde at 28 h.p.f.

### Imaging

Sections were analysed with a DM or confocal microscope, whereas whole embryos were observed with a stereomicroscope (Leica Microsystems). Digital images were obtained using DFC500, DFC350 FX cameras (Leica) or confocal microscopy (Zeiss) and processed with Photoshop CS5 or ImageJ (Fiji) software.

### Quantification and statistical analysis

For *pax2.1* quantifications, embryos were mounted in glycerol 85% and photographed in lateral views at × 160 to quantify the area of the *pax2.1*-positive optic stalk domain. Comparison of *nkx2.2* and *nkx2.1*-positive domains in control and morphant embryos was performed using dorsal view images of whole embryos and determining the mediolateral extent of the signal. Quantitative analysis of Shh levels in electroporated cells was performed using confocal images of transversal sections of control and experimental embryos at the level of the optic stalk. In each one of the selected sections (one section per embryo, 7–14 embryos per case were analysed), Shh levels were measured in two adjacent regions (proximal and distal) outside of the endogenous Shh source. These values were plotted separately. Quantitative analysis of width of the basal end-foot area was performed using confocal images of Cherry, Cdon or deleted derivatives overexpressing cells. Quantifications were performed using ImageJ (NIH). Data analyses were performed with the IBM SPSS statistic software. All the data sets presented a normal distribution (Kolmogorov–Smirnov test). Analysis of the data was performed using *t*-test for two groups and analysis of variance for larger groups. Statistical differences between pools of treated (MO, drugs, overexpression) and control embryos were determined with the Pearson’s *χ*^2^ test. In all graphs the error bars indicate s.e.m.

## Author contributions

M.J.C. participated in all experiments presented in this work, analysed data and contributed to experimental design and manuscript writing. L.S.-A. performed studies in chick embryos and analysed the data. A.S. performed histological and confocal work and analysed the data. C.S.-C. performed experiments related to Boc function. S.W.W., G.G. and I.G. contributed to experimental design and revised the manuscript. Artwork was generated by M.J.C. and L.S.A. P.B. directed the work, analysed data and wrote the manuscript.

## Additional information

**How to cite this article:** Cardozo, M-J. *et al.* Cdon acts as a Hedgehog decoy receptor during proximal-distal patterning of the optic vesicle. *Nat. Commun.* 5:4272 doi: 10.1038/ncomms5272 (2014).

## Supplementary Material

Supplementary Information Supplementary Figures 1-13, Supplementary Table 1 and Supplementary References
