# Supplementary material for: Cdon acts as a Hedgehog decoy receptor during proximal-distal patterning of the optic vesicle
Source: Nat Commun. 2014 Jul 8;5:4272. doi: 10.1038/ncomms5272 (PMC4102123; doi:10.1038/ncomms5272)
Supplement: Supplementary Information — Supplementary Figures 1-13, Supplementary Table 1 and Supplementary References [file ncomms5272-s1.pdf]

SUPPLEMENTARY INFORMATION

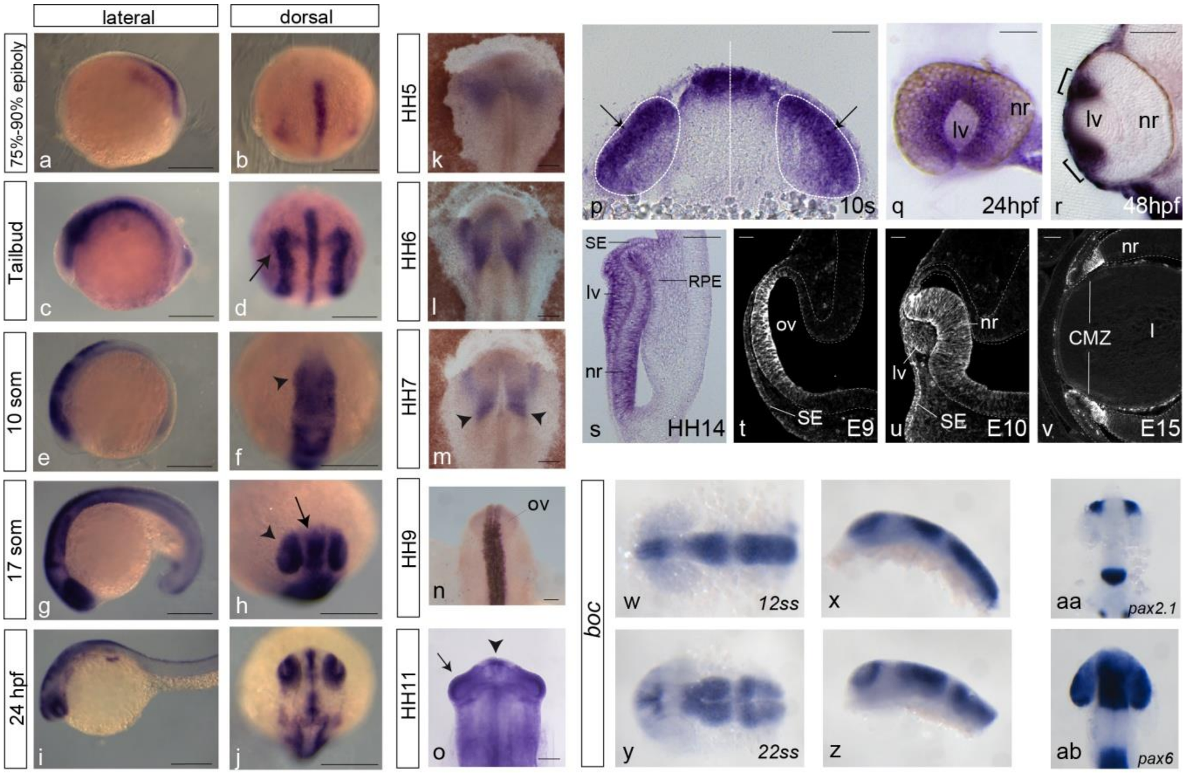

Supplementary Figure 1. *Cdon* expression in vertebrate embryos. Lateral (**a-i**) and dorsal (**b-j**) views of zebrafish and ventral (**k-m**) and dorsal (**n**, **o**) views of chick embryos at different developmental stages hybridized *in toto* with a *Cdon* specific probe and immunohistochemistry of Cdon distribution in the developing mouse embryonic eye (**t-v**). The zebrafish distribution of *boc* (**w-z**), *pax2.1* (**aa**) and *pax6* (**ab**) is shown for comparison with embryos hybridized *in toto* with probes specific for *cdon* at different stages of development. Images in (**p, r**) are coronal cryostat sections of zebrafish embryos hybridized *in toto*. In zebrafish at 75-90% epiboly *cdon* transcripts are detected in the ventral midline (**a, b**). At tailbud stage *cdon* mRNA localizes to the presumptive neural crest (**c, d** arrow) and at 10-17 somite stages in the optic vesicles (**f, h** arrowhead**)**, prechordal plate, telencephalon (**h**, arrow) and dorsal neural tube (**e-h**). At 24 hpf *cdon* expression becomes restricted to the retina and the dorsal neural tube (**i, j**). In chick embryos, *Cdon* expression is first detected at the Hensen´s node (**k**), and later in the presumptive neural crest (**l-n**), somites (**m** arrowheads) and CNS (**n**). At HH11 *Cdon* mRNA localizes to the optic vesicles and dorsal CNS (**o,** arrow and arrowhead respectively). *Cdon* expression is restricted to regions of the telencephalon and the presumptive retinal domain of the optic vesicle at 10ss stage (**p**) to finally restricting to the ciliary margin zone (CMZ) (see brackets in **q, r**). *Cdon* expression in the developing eye is conserved in chick (**s**) and mouse embryos (**t-v**). Note that *boc* is not expressed in the optic vesicle (**w, x**) or the optic cup (**y**, **z**). The white dashed lines in **p** indicate the midline and the optic vesicles. The white dashed line in **t-v** delineates the neural epithelium. CMZ, ciliary marginal zone; l, lens; lv, lens vesicle; nr, neural retina; ov, optic vesicle; RPE, retinal pigmented epithelium; SE, surface ectoderm. Scale bars: 200 µm.

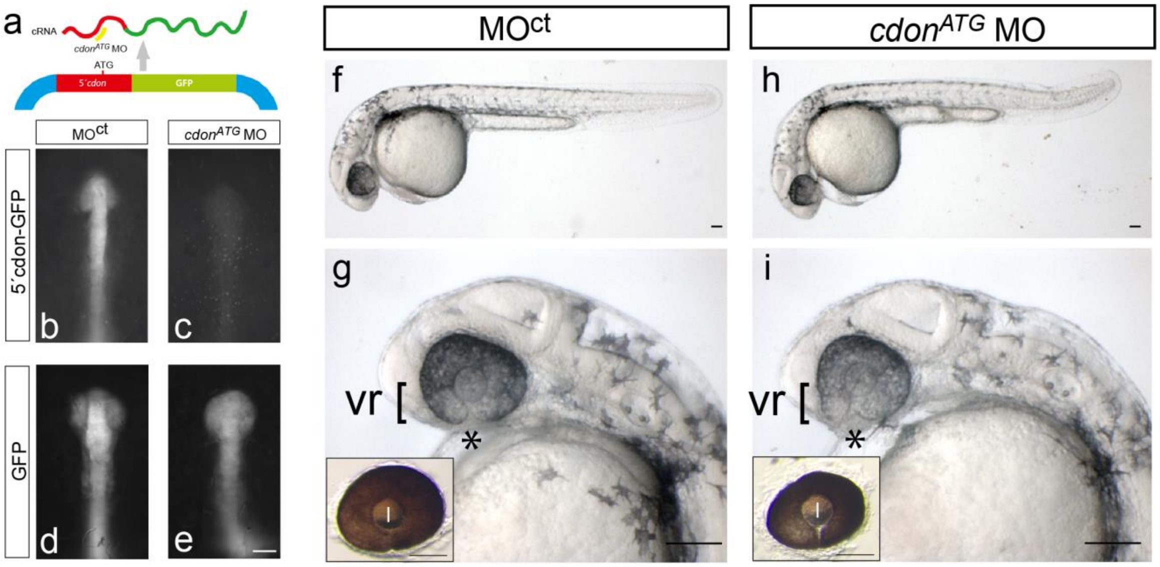

Supplementary Figure 2. *cdon*^{ATG} MO efficiently interferes with *cdon* expression inducing morphants that present defects in the ventral region of the eye. a) Schematic diagram of the strategy used to test the specificity of an antisense morpholino (MO) designed against the translation start site sequence of *cdon* (*cdon*^{ATG}). This MO or a standard control MO (*MO*^{ct}) were co-injected into medaka fish embryos with the cRNA of a reporter construct (5´*cdon*-GFP) carrying the zebrafish *cdon* 5´ sequence upstream of the *gfp* sequence. Translation of this construct (evident by expression of GFP) was efficiently knocked down by *cdon*^{ATG} MO (**c**) (25/25 embryos) but not by the *MO*^{ct} (**b**) (20/20 embryos). None of the two MO interfered with the translation of the GFP alone (**d, e**) (23/23 embryos). Bright field lateral (**f-i**) views of embryos injected with *MO*^{ct} (**f, g**) or *cdon*^{ATG} MO (**h, i**). *cdon*^{ATG} MO (**h, i**) but not *MO*^{ct} (**f, g**) injected embryos are characterized by alteration of the ventral retina (vr) at 28 hpf. The asterisks indicate the optic fissure in *cdon*^{ATG} morphant (**i**)**,** which remains open at 36 hpf (compare insets in **g, i**). l, lens; vr, ventral retina. Scale bars: 40 µm (**b-e**). Scale bars: 100 µm (**f-i**).

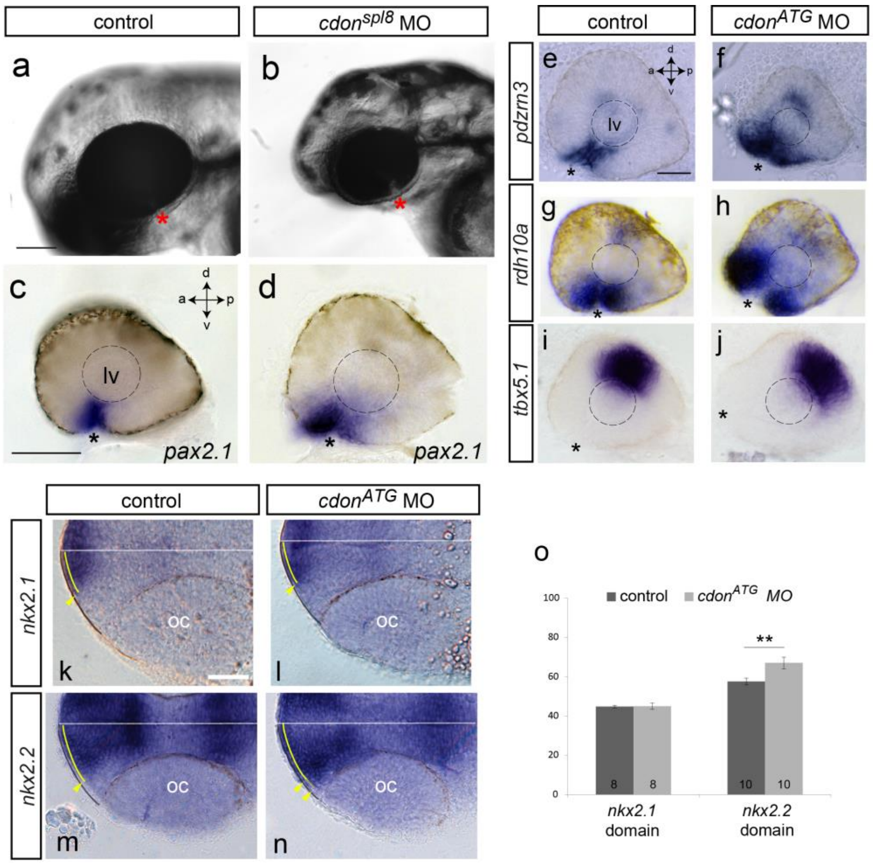

Supplementary Figure 3. ***cdon^{spl8}*** **splicing MO reproduces the phenotype observed in** ***cdon^{ATG}*** **morphants, which display an expansion of ventral markers.** Bright field, lateral images of 48 hpf control (**a**) and *cdon^{spl8}* morphant characterized by the presence of coloboma (**b**). Lateral views of the eyes of control and *cdon^{spl8}* morphants hybridized *in toto* with probes for *pax2.1* (**c, d**) (17/40 embryos). Note that in *cdon^{spl8}* morphants *pax2.1* expression is expanded in comparison to controls (**c, d**). Lateral views of the eyes of control and *cdon^{ATG}* morphants hybridized *in toto* with probes for the ventral retinal markers *pdzrn3* (**e**, **f**) and *rdh10a* (**g**, **h**) or the dorsal retinal marker *tbx5.1* (**i**, **j**). In *cdon^{ATG}* morphants the ventral retina markers *pdzrn3* (28/54 embryos) and *rdh10a* (32/44 embryos) are also expanded in comparison to controls (**e-h**) whereas *tbx5.1* (62/62 embryos) shows no evident changes in comparison to controls (**i, j**). The dashed lines mark the lens, and the asterisks indicate the position of the optic fissure. (**k-n**) Horizontal cryostat sections of control and *cdon^{ATG}* morphant embryos hybridized with probes for *nkx2.1* and *nkx2.2* at 26hpf**.** The dotted black lines indicate the distance from the midline to the optic cup. The yellow lines depict the extent of gene expression in the medio-lateral direction and the yellow arrowheads indicate the most distal/lateral position of the positive signal. The dotted yellow line in **n** depicts the expansion of *nkx2.2* expression observed in *cdon^{ATG}* embryos. **o**) Quantitative analysis of the *nkx2.1* and *nkx2.2* anterior expression domains. Note that *nkx2.2* expression in morphants is significantly different from that of controls (**$p < 0.01$; Student's *t*-test). The number of embryos analysed in each case is as follow: *nkx2.1*, n=8 for both control and MO injected embryos; *nkx2.2*, n=10 for both control and MO injected embryos). This information is also indicated in each column Error bars in **o** represent s.e.m. a, anterior; d, dorsal; lv, lens vesicle; oc, optic cup; p, posterior; v, ventral. Scale bars: 100 µm.

a)

MHPDLGPLCTLLYVTLTILCSSVSSDLAPYFTSEPLSAVQKLGGPVVLHCSAQPVTTRISWLHNGKTLDGNLEHVKIHQGTLTILSLNSSLLGYYQCLANNSIGAIVSGPATVSVAVLGDFG
SSTKHVITAEEKSAGFIGCRVPESNPKAEVRYKIRGKWLEHSTENYLILPSGNLQILNVSLEDKGSYKCAAYNPVTHQLKVEPIGRKLLVSRPSSDDVHILHPTHSQALAVLSRSPVTLECV
VSGVPAPQVYWLKDGQDIAPGSNWRRLYSHLATDSVDPADSGNYSCMAGNKSGDVKYVTYMVNVLEHASISKGLQDQIVSLGATVHFTCDVHGNPAPNCTWFHNAQPIHPSARHLTAGNGLK
ISGVTVEDVGMYQCVADNGIGFMHSTGRLEIENDGGFKPVIITAPVSAKVADGDFVTLSCNASGLPVPVIRWYDSHGLITSHPSQVLRSKSRKSQLSRPEGLNLEPVYFVLSQAGASSLHIQ
AVTQEHAGKYICEAANEHGTTQAEASLMVVPFETNTKAETVTLPDAAQNDDRSKRDGSETGLLSSFPVKVHPSAVESAPEKNASGISVPDAPIILSPPQTHTPDTYNLVWRAGKDGGLPINA
YFVKYRKLDDGVGMLGSWHTVRVPGSENELHLAELEPSSLYEVLMVARSAAGEGQPAMLTFRTSKEKTASSKNTQASSPPVGIPKYPVVSEAANNNFGVVLTDSSRHSGVPEAPDRPTISTA
SETSVYVTWIPRANGGSPITAFKVEYKRMRTSNWLVAAEDIPPSKLSVEVRSLEPGSTYKFRVIAINHYGESFRSSASRPYQVVGFPNRFSSRPITGPHIAYTEAVSDTQIMLKWTYIPSSN
NNTPIQGFYIYYRPTDSDNDSDYKRDVVEGSKQWHMIGHLQPETSYDIKMQCFNEGGESEFSNVMICETKVKRVPGASEYPVKDLSTPPNSLGSGGNVGPATSPARSSDMLYLIVGCVLGVM
VLILMVFIAMCLWKNRQQNTIQKYDPPGYLYQGSDMNGQMVDYTTLSGASQINGNVHGGFLTNGGLSSGYSHLHHKVPNAVNGIVNGSLNGGLYSGHSNSLTRTHVDFEHPHHLVNGGGMYT
AVPQIDPLECVNCRNCRNNNRCFTKTNSTFSSSPPPVVPVVAPYPQDGLEMKPLSHVKVPVCLTSAVPDCGQLPEESVKDNVEPVPTQRTCCQDIVNDVSSDGSEDPAEFSRGQEGMINLRI
PDHLQLAKSCVWEGDSCAHSETEINIVSWNALILPPVPEGCAEKTMWSPPGIPLDSPTEVLQQPRET

b)

MHPDLGPLWTLLYVLVILCSSVSSDLAPYFISEPLSAVQKLGRPVVLHCSAKPVTARISWLHNGKRLDRNTEQIKIHRGTLTILSLNPSLSGCYQCVANNSVGAVVSGPATVSAAALGDFDS
STMHVITAEEKNTGFIGCRVPESNPKAEVRYKIRGKWLKHSTGNYIILPSGNLQVLNVSSKDKGSYKCAAYNPVTSELKVEPTGRKLLVSRPSSNGFHILHPALSQALAVLPHSPVTLECVV
SGVPASQVYWLKDGQDAVAGSNWRRLYSHLATASIDPADSGNYSCVVGNKSGDVKHVTYMVNVLEHASISKGLHDQKVSLGATVHFTCDVHGNPAPNRTWFHNAQPIHPSSRHLTEGNVLKI
TRVVMEDSGLYQCVADNGIGFMQSTGRLQIEQDSGWKPVIVTAPANIEVMDGDFVTLSCNATGVPVPVIHWYGRHGLITSHPSQVLRSKPRKSHLFRPGDLDLEPVYLIMSQAGSSSLSIQA
VTLEHAGKYTCEATNKHGSTQSEAFLTVVPFETNTKAESVTPSEASQNDERDPQDGSESSLLNLFPVKVHPSGVELPAERNASVPDAPNILSPPQTHMPDTYNLVWRAGRDGGMPINAYFVK
YRKLDDGSGAVGSWHTVRVPGSENELHLTELEPSSLYEVLMVARSAVGEGQPAMLTFRTSKEKMASSKNTQASFPPVGVPKRPVTAEASNSNFGVVLTDSSRHSGVPEAPDRPTISMASETS
VYVTWIPRANGGSPITAFKVEYKRMRTSDWLVAAEDIPPSKLSVEVRSLEPGSIYKFRVIAINHYGESFRSSASRPYQVAGFPNRFSNRPITGPHIAYTEAVSDTQIMLKWTYVPSSNNNTP
IQGFYIYYRPTDSDNDSDYKRDVVEGSKQWHTIGHLQPETSYDIKMQCFNEGGESEFSNVMICETKVKRVPGASDYPVKELSTPPSSSGNAGNVGPATSPARSSDMLYLIVGCVLGVMVLIL
MVFIALCLWKSRQQSTIQKYDPPGYLYQGSEINGQMVEYTTLSGAARINGSVHGGFLSNGCSHLHHKGPSGVNGTLSGNINGGLYSAHTNSLTRACVEFEHPHHLVNSGGVYTAVPQMDPLE
CINCRNCRNNNRCFTKTNSPLPVVPVVASYPQGGLEMKPLNAMKVPVCPASTVPDHGQLPDDCVKDSVAPIPTQHTCCQDNISDINSDSTEDTAEFSRGDSSGHSEAEDKVFSWNPLILSPV
LEDCGEKTARSPPGPPLDGLSVVLQQAQET

Supplementary Figure 4. Sequences of the human and mouse Cdon protein. Cdon protein sequences from *Homo sapiens* (Q4KMG0; **a**) and *Mus musculus* (Q32MD9; **b**) as well as the position and sequence of the fibronectin type-III 2 (blue) and 3 (green) domains were obtained from Uniprot database[1].

```
Cdon_Danio_rerio          MEDGGLRLLSAVLCVCHTLLLNCPTVLSFSFRAEPLSAILKQGSSVHLHCTTHPATARIS
FnIII_2_Mus_musculus      ------------------------------------------------------------
FnIII_2_Homo_sapiens      ------------------------------------------------------------

Cdon_Danio_rerio          WLFQGQPLDPSHHSGVELSQDSLSLSNLQPALTGSYQCSARSETGSIISRHARVTIADIE
FnIII_2_Mus_musculus      ------------------------------------------------------------
FnIII_2_Homo_sapiens      ------------------------------------------------------------

Cdon_Danio_rerio          EFAETHRRSFTVNKGDTAVIECPLPRSNPPALPRFRIRGKWLEQSTDEYLILPSGNLQIV
FnIII_2_Mus_musculus      ------------------------------------------------------------
FnIII_2_Homo_sapiens      ------------------------------------------------------------

Cdon_Danio_rerio          SVSSEHQGMYKCGAYNPLTRETRVEAHGTKLLVKDSESSSPVRIVYPITPRSLTVDQSGS
FnIII_2_Mus_musculus      ------------------------------------------------------------
FnIII_2_Homo_sapiens      ------------------------------------------------------------

Cdon_Danio_rerio          LTLECVVSGSLSSKVKWMKNGAELSLSSKRMLSHSNLVLNDIQPGDGGHYSCSVPTDRGA
FnIII_2_Mus_musculus      ------------------------------------------------------------
FnIII_2_Homo_sapiens      ------------------------------------------------------------

Cdon_Danio_rerio          VVSVNYTVNVLAHVSILRGLSDQAAVAGSSVRFTCAASGNPTPNITWLLNAAPLSSSPRL
FnIII_2_Mus_musculus      ------------------------------------------------------------
FnIII_2_Homo_sapiens      ------------------------------------------------------------

Cdon_Danio_rerio          KISGTSLLISSTTLQDQGIYQCMFDNGISSAQSTGRLSIQSEPQSSSISAVPVKTQPSVH
FnIII_2_Mus_musculus      ------------------------------------------------------------
FnIII_2_Homo_sapiens      ------------------------------------------------------------

Cdon_Danio_rerio          PIQSDEGDELFLSMGEAALGETVGPPTERIGDRPTPEAPIIISPPQTHKPNMYDLEWRAG
FnIII_2_Mus_musculus      ------------------------------------------------------------
FnIII_2_Homo_sapiens      ------------------------------------------------------------

Cdon_Danio_rerio          RDWGIAIIAYFVKYRKVDDMGNVVGSWHTVRVPGSEKSLPLSELEPSSLYEVLMVARSAA
FnIII_2_Mus_musculus      ------------------------------------------------------------
FnIII_2_Homo_sapiens      ------------------------------------------------------------

Cdon_Danio_rerio          GEGQPAMLTFRTGKERASPNKNPSKAPIVSLPEKTPEDKTTNTYYGVVIHDRVPEAPDRP
FnIII_2_Mus_musculus      -----------------------------------------------------PEAPDRP
FnIII_2_Homo_sapiens      -----------------------------------------------------PEAPDRP
                                                                               *******

Cdon_Danio_rerio          TISMATESSVYVTWIPRANGGSPITAFRVEYRKQGRNGDWIIAADNISPLKLSVEVRNLE
FnIII_2_Mus_musculus      TISMASETSVYVTWIPRANGGSPITAFKVEYKRM-RTSDWLVAAEDIPPSKLSVEVRSLE
FnIII_2_Homo_sapiens      TISMASETSVYVTWIPRANGGSPITAFKVEYKRM-RTSDWLVAAEDIPPSKLSVEVRSLE
                          *****:*:*******************:***::  *..**::**::* * *******.**

Cdon_Danio_rerio          PGSTYRFRVIAMNNYGESPASATSRPYQVSMSSSPVSNRPVTGPHISSTDAVSDTQILLR
FnIII_2_Mus_musculus      PGSIYKFRVIAINHYGESFRSSASRPY---------------------------------
FnIII_2_Homo_sapiens      PGSIYKFRVIAINHYGESFRSSASRPY---------------------------------
                          *** *:*****:*.****  *::****

Cdon_Danio_rerio          WTYTPSSNNNTPIQGFYIYYRPTDSDNDSDYKKDVVEGFKFWHMIGELQPETSYDIKMQC
FnIII_2_Mus_musculus      ------------------------------------------------------------
FnIII_2_Homo_sapiens      ------------------------------------------------------------

Cdon_Danio_rerio          YNDGGESEYSNVMICETKARQPPGVPSLRPITPPGFYPADTPSQPGGLLYLIVGCVLGVM
FnIII_2_Mus_musculus      ------------------------------------------------------------
FnIII_2_Homo_sapiens      ------------------------------------------------------------
Cdon_Danio_rerio          VLILLVFIVMCLWRNRQQNSMHKYDPPNYIYQSAEMNGHVLDYSALPGSSHVNGSVHTGC
FnIII_2_Mus_musculus      ------------------------------------------------------------
FnIII_2_Homo_sapiens      ------------------------------------------------------------

Cdon_Danio_rerio          GHTAPMMPQTCHHLHHKLPNGLALLNGSGGLYPAGHPHAHDTSLHQNNMEYEHPSPHHLH
FnIII_2_Mus_musculus      ------------------------------------------------------------
FnIII_2_Homo_sapiens      ------------------------------------------------------------

Cdon_Danio_rerio          NGGGIYTALPQNDSSDCMSCQNFCNNNRCYTKTNGTFSGGTLPLMHRVAARQPDGLEMMP
FnIII_2_Mus_musculus      ------------------------------------------------------------
FnIII_2_Homo_sapiens      ------------------------------------------------------------

Cdon_Danio_rerio          LNPILSRCHGRDSPQLNGCQDRDSVQQAEEGNVPPLSHNSPCLPVETKSSLEQQRGVQQV
FnIII_2_Mus_musculus      ------------------------------------------------------------
FnIII_2_Homo_sapiens      ------------------------------------------------------------

Cdon_Danio_rerio          QHEDSEGPVVCWERLGLDLDCKEKTAWISTGSLTGDLIQPTVQEI
FnIII_2_Mus_musculus      ---------------------------------------------
FnIII_2_Homo_sapiens      ---------------------------------------------
```

Supplementary Figure 5. Alignment of the mouse and human Cdon FNIII 2 sequences with the Cdon zebrafish sequence (Uniprot, Q1L8D0) using Clustal Omega[2, 3].

```
Cdon_Danio_rerio          MEDGGLRLLSAVLCVCHTLLLNCPTVLSFSFRAEPLSAILKQGSSVHLHCTTHPATARIS
FnIII_3_Mus_musculus      ------------------------------------------------------------
FnIII_3_Homo_sapiens      ------------------------------------------------------------

Cdon_Danio_rerio          WLFQGQPLDPSHHSGVELSQDSLSLSNLQPALTGSYQCSARSETGSIISRHARVTIADIE
FnIII_3_Mus_musculus      ------------------------------------------------------------
FnIII_3_Homo_sapiens      ------------------------------------------------------------

Cdon_Danio_rerio          EFAETHRRSFTVNKGDTAVIECPLPRSNPPALPRFRIRGKWLEQSTDEYLILPSGNLQIV
FnIII_3_Mus_musculus      ------------------------------------------------------------
FnIII_3_Homo_sapiens      ------------------------------------------------------------

Cdon_Danio_rerio          SVSSEHQGMYKCGAYNPLTRETRVEAHGTKLLVKDSESSSPVRIVYPITPRSLTVDQSGS
FnIII_3_Mus_musculus      ------------------------------------------------------------
FnIII_3_Homo_sapiens      ------------------------------------------------------------

Cdon_Danio_rerio          LTLECVVSGSLSSKVKWMKNGAELSLSSKRMLSHSNLVLNDIQPGDGGHYSCSVPTDRGA
FnIII_3_Mus_musculus      ------------------------------------------------------------
FnIII_3_Homo_sapiens      ------------------------------------------------------------

Cdon_Danio_rerio          VVSVNYTVNVLAHVSILRGLSDQAAVAGSSVRFTCAASGNPTPNITWLLNAAPLSSSPRL
FnIII_3_Mus_musculus      ------------------------------------------------------------
FnIII_3_Homo_sapiens      ------------------------------------------------------------

Cdon_Danio_rerio          KISGTSLLISSTTLQDQGIYQCMFDNGISSAQSTGRLSIQSEPQSSSISAVPVKTQPSVH
FnIII_3_Mus_musculus      ------------------------------------------------------------
FnIII_3_Homo_sapiens      ------------------------------------------------------------

Cdon_Danio_rerio          PIQSDEGDELFLSMGEAALGETVGPPTERIGDRPTPEAPIIISPPQTHKPNMYDLEWRAG
FnIII_3_Mus_musculus      ------------------------------------------------------------
FnIII_3_Homo_sapiens      ------------------------------------------------------------

Cdon_Danio_rerio          RDWGIAIIAYFVKYRKVDDMGNVVGSWHTVRVPGSEKSLPLSELEPSSLYEVLMVARSAA
FnIII_3_Mus_musculus      ------------------------------------------------------------
FnIII_3_Homo_sapiens      ------------------------------------------------------------

Cdon_Danio_rerio          GEGQPAMLTFRTGKERASPNKNPSKAPIVSLPEKTPEDKTTNTYYGVVIHDRVPEAPDRP
FnIII_3_Mus_musculus      ------------------------------------------------------------
FnIII_3_Homo_sapiens      ------------------------------------------------------------

Cdon_Danio_rerio          TISMATESSVYVTWIPRANGGSPITAFRVEYRKQGRNGDWIIAADNISPLKLSVEVRNLE
FnIII_3_Mus_musculus      ------------------------------------------------------------
FnIII_3_Homo_sapiens      ------------------------------------------------------------

Cdon_Danio_rerio          PGSTYRFRVIAMNNYGESPASATSRPYQVSMSSSPVSNRPVTGPHISSTDAVSDTQILLR
FnIII_3_Mus_musculus      ---------------------------------------PITGPHIAYTEAVSDTQIMLK
FnIII_3_Homo_sapiens      ---------------------------------------PITGPHIAYTEAVSDTQIMLK
                                                                 *:*****: *:*******:*:

Cdon_Danio_rerio          WTYTPSSNNNTPIQGFYIYYRPTDSDNDSDYKKDVVEGFKFWHMIGELQPETSYDIKMQC
FnIII_3_Mus_musculus      WTYVPSSNNNTPIQGFYIYYRPTDSDNDSDYKRDVVEGSKQWHTIGHLQPETSYDIKMQC
FnIII_3_Homo_sapiens      WTYIPSSNNNTPIQGFYIYYRPTDSDNDSDYKRDVVEGSKQWHMIGHLQPETSYDIKMQC
                          *** ****************************:***** * ** **.*************

Cdon_Danio_rerio          YNDGGESEYSNVMICETKARQPPGVPSLRPITPPGFYPADTPSQPGGLLYLIVGCVLGVM
FnIII_3_Mus_musculus      FNEGGESEFSNVMICET-------------------------------------------
FnIII_3_Homo_sapiens      FNEGGESEFSNVMICET-------------------------------------------
                          :*:*****:********

Cdon_Danio_rerio          VLILLVFIVMCLWRNRQQNSMHKYDPPNYIYQSAEMNGHVLDYSALPGSSHVNGSVHTGC
FnIII_3_Mus_musculus      ------------------------------------------------------------
FnIII_3_Homo_sapiens      ------------------------------------------------------------

Cdon_Danio_rerio          GHTAPMMPQTCHHLHHKLPNGLALLNGSGGLYPAGHPHAHDTSLHQNNMEYEHPSPHHLH
FnIII_3_Mus_musculus      ------------------------------------------------------------
FnIII_3_Homo_sapiens      ------------------------------------------------------------

Cdon_Danio_rerio          NGGGIYTALPQNDSSDCMSCQNFCNNNRCYTKTNGTFSGGTLPLMHRVAARQPDGLEMMP
FnIII_3_Mus_musculus      ------------------------------------------------------------
FnIII_3_Homo_sapiens      ------------------------------------------------------------

Cdon_Danio_rerio          LNPILSRCHGRDSPQLNGCQDRDSVQQAEEGNVPPLSHNSPCLPVETKSSLEQQRGVQQV
FnIII_3_Mus_musculus      ------------------------------------------------------------
FnIII_3_Homo_sapiens      ------------------------------------------------------------

Cdon_Danio_rerio          QHEDSEGPVVCWERLGLDLDCKEKTAWISTGSLTGDLIQPTVQEI
FnIII_3_Mus_musculus      ---------------------------------------------
FnIII_3_Homo_sapiens      ---------------------------------------------
```

Supplementary Figure 6. Alignment of the mouse and human Cdon FNIII 2 sequences with the Cdon zebrafish sequence (Uniprot, Q1L8D0) using Clustal Omega[2, 3].

MEDGGLRLLSAVLCVCHTLLLNCPTVLSFSFRAEPLSAILKQGSSVHLHCTTHPATARISWLFQGQPLDPSHHSGVELSQDSLSLSNLQPALTGSYQCSARSETGSIISRHARVTIADIEEF
AETHRRSFTVNKGDTAVIECPLPRSNPPALPRFRIRGKWLEQSTDEYLILPSGNLQIVSVSSEHQGMYKCGAYNPLTRETRVEAHGTKLLVKDSESSSPVRIVYPITPRSLTVDQSGSLTLE
CVVSGSLSSKVKWMKNGAELSLSSKRMLSHSNLVLNDIQPGDGGHYSCSVPTDRGAVVSVNYTVNVLAHVSILRGLSDQAAVAGSSVRFTCAASGNPTPNITWLLNAAPLSSSPRLKISGTS
LLISSTTLQDQGIYQCMFDNGISSAQSTGRLSIQSEPQSSSISAVPVKTQPSVHPIQSDEGDELFLSMGEAALGETVGPPTERIGDRPTPEAPIIISPPQTHKPNMYDLEWRAGRDWGIAII
AYFVKYRKVDDMGNVVGSWHTVRVPGSEKSLPLSELEPSSLYEVLMVARSAAGEGQPAMLTFRTGKERASPNKNPSKAPIVSLPEKTPEDKTTNTYYGVVIHDRVPEAPDRPTISMATESSV
YVTWIPRANGGSPITAFRVEYRKQGRNGDWIIAADNISPLKLS**V**EVRNLEPGS**T**YRFRVIAMNNYGESPASATSRPYQVSMSSSPVSNRPVTGPHISSTDAVSDTQILLRWTYTPSSNNNTP
IQGFYIYYRPT**D**S**D**NDSDYKKDVVEGFKFWHMIGELQP**E**TSY**D**IKMQCYNDGGESEYSNVMIC**E**TKARQPPGVPSLRPITPPGFYPADTPSQPGGLLYLIVGCVLGVMVLILLVFIVMCLWR
NRQQNSMHKYDPPNYIYQSAEMNGHVLDYSALPGSSHVNGSVHTGCGHTAPMMPQTCHHLHHKLPNGLALLNGSGGLYPAGHPHAHDTSLHQNNMEYEHPSPHHLHNGGGIYTALPQNDSSD
CMSCQNFCNNNRCYTKTNGTFSGGTLPLMHRVAARQPDGLEMMPLNPILSRCHGRDSPQLNGCQDRDSVQQAEEGNVPPLSHNSPCLPVETKSSLEQQRGVQQVQHEDSEGPVVCWERLGLD
LDCKEKTAWISTGSLTGDLIQPTVQEI

Supplementary Figure 7. Cdon *Danio rerio* sequence. Sequence of the zebrafish Cdon protein (Uniprot, Q1L8D0), in which the residues of the deduced fibronectin type-III(2) and fibronectin type-III(3) are indicated in blue and green, respectively. The homolog residues relevant for Ptch binding[4] are highlighted in light blue, those relevant for Shh binding[5] in red.

a)TATCCATCCTGCGAGGTTTGTCAGATCAGGCTGCAGTTGTTGGATTCTCTGTGAGATTTACCTGTGCTGCCAGTGGCAATCCAACCCCCAACATCACATGGCTCCTTAACGCTGCCCCTT
TATCTTCTTCACCCCGCCTCAAAATCTCTGGCACCTCCCTTCACATCTCGTCAACAACGCTTCAAGATCAGGGAATCTACCAGTGCATGTTTGATAATGGCATTAGCTCTGCACAATCCACA
GGAAGACTCAGTATCCAATCAGAACCACAGTCTAGTTCCATATCTGCAGTTCCAGTGAAGACGCAACCTTCTGTTCACCCAATCCAGAGTGATGAGGGTGATGAACTGTTCCTCTCCATGGG
GGAGGCGGCGCTTGGAGAAACTGTAGGACCGCCCACCGAGAGAATCGGCGACAGACCCACACCAGAAGCACCAATCATAATCAGCCCTCCTCAAACACACAAACCCAACATGTACGATCTGG
AGTGGAGAGCAGGCCGAGACTGGGGCATCGCTATCATCGCATACTTTGTCAAATATCGCAAGGTGGATGATATGGGAAATGTGGTGGGGAGCTGGCACACGGTGCGGGTACCCGGCAGCGAG
AAGTCTCTGCCCTTGTCAGAGCTGGAGCCGTCCAGCCTGTATGAGGTC

b)TATCCATCCTGCGAGGTTTGTCAGATCAGGCTGCAGTTGTTGGATCCTCTGTGAGATTTACCTGTGCTGCCAGTGGCAATCCAACCCCCAACATCACATGACTCCTTAACGCTGCCCCTT
TATCTTCTTCACCCCGCCTCAAAATCTCTGGCACCTCCCTTCACGTCTCGTCAACAACGCTTCAAGATCAGGGAATCTACCAGTGCATGTTTGATAATGGCATTAGCTCTGCACAATCCACA
GGAAGACTCAGTATCCAATCAGAACCACAGTCTAGTTCCATATCTGCAGTTCCAGTGAAGACGCAACCTTCTGTTCACCCAATCCAGAGTGATGAGGGTGATGAACTGTTCCTCTCCATGGG
GGAGGCGGCGCTTGGAGAAACTGTAGGACCGCCCACCGAGAGAATCGGCGACAGACCCACACCAGAAGCACCAATCATAATCAGCCCTCCTCAAACACACAAACCCAACATGTGGATGATAT
GGGAAATGTGGTGGGGAGCTGGCACACGGTGCGGGTACCCGGCAGCGAGAAGTCTCTGCCCTTGTCAGAGCTGGAGCCGTCCAGCCTGTATGAGGTC

c)TATCCATCCTGCGAGGTTTGTCAGATCAGGCTGCAGTTGCTGGATCCTCTGTGAGATTTACCTGTGCTGCCAGTGGCAATCCAACCCCCAACATCACATGGCTCCTTAACGCTGCCCCTT
TATCTTCTTCACCCCGCCTCAAAATCTCTGGCACCTCCCTTCTCATCTCTTCAACAACGCTTCAAGATCAGGGAATCTACCAGTGCATGTTTGATAATGGCATTAGCTCTGCACAATCCACA
GGAAGACTCAGTATCCAATCAGAACCACAGTCTAGTTCCATATCTGCAGTTCCAGTGAAGACGCAACCTTCTGTTCACCCAATCCAGAGTGATGAGGGTGATGAACTGTTCCTCTCCATGGG
GGAGGCGGCGCTTGGAGAAACTGTGGATGATATGGGAAATGTGGTGGGGAGCTGGCACACGGTGCGGGTACCCGGCAGCGAGAAGTCTCTGCCCTTGTCAGAGCTGGAGCCGTCCAGCCTGT
ATGAGGTC

d)TATCCATCCTGCGAGGTTTGTCAGATCAGGCTGCAGTTGCTGGATCCTCTGTGAGATTTACCTGTGCTGCCAGTGGCAATCCAACCCCCAACATCACATGGCTCCTTAACGCTGCCCCTT
TATCTTCTTCACCCCGCCTCAAAATCTCTGGCACCTCCCTTCTCATCTCTTCAACAACGCTTCAAGATCAGGGAATCTACCAGTGCATGTTTGATAATGGCATTAGCTCTGCACAATCCACA
GGAAGACTCAGTATCCAATCAGGTGGATGATATGGGAAATGTGGTGGGGAGCTGGCACACGGTGCGGGTACCCGGCAGCGAGAAGTCTCTGCCCTTGTCAGAGCTGGAGCCGTCCAGCCTGT
ATGAGGTC

e)ACACTCCATCCAGCAATAACAACACTCCCATCCAGGGCTTCTACATCTACTATCGGCCAACAGACAGCGACAATGACAGCGACTACAAGAAAGATGTGGTGGAAGGCTTCAAATTCTGGC
ACATGATTGGTGAACTCCAACCCGAGACATCTTATGACATCAAGATGCAGTGCTATAATGACGGCGGCGAGAGCGAATACAGTAACGTCATGATCTGCGAGACCAAAGCCCGGCAGCCTCCA
GGTGTGCCTAGCCTGCGTCCTATCACTCCTCCAGGCTTCTATCCTGCTGATACACCCAGCCAGCCGGGCGGCCTGCTCTACCTGATCGTGGGCTGTGTTCTGGGCGTCATGGTGCTCATTCT
GCTTGTCTTCATCGTCATGTGCTTATGGAGGA

f)ACACTCCATCCAGCAATAACAACACTCCCATCCAGGGCTTCTACATCTACTATCGGCCAACAGACAGCGACAATGACAGCGACTACAAGAGAGATGTGGTGGAAGCCCGGCAGCCTCCAG
GTGTGCCTAGCCTGCGTCCCATCACTCCTCCAGGCTTCTATCCTGCTGATACACCCAGCCAGCCGGGCGGCCTGCTCTACCTGATCGTGGGCTGTGTTCTGGGCGTCATGGTGCTCATTCTG
CTTGTCTTCATCGTCATGTGCTTATGGAGGA

g)CCAATAAGAATCCCTCCAAAGCTCCAATTGTGTCTCTACCAGAGAAAACTCCAGAAGATAAAACTACTAACACTTATTATGGAGTCGTCATACATGACAGAGTTCCTGAGGCTCCAGACC
GCCCCACAATCTCAATGGCGACAGAAAGTTCTGTTTACGTCACATGGATACCTCGGGCCAATGGTGGCTCTCCGATCACTGCATTTCGCGTGGAGTACAGAAAACAGGGGCGTAATGGAGAC
TGGATCATTGCAGCTGATAACATCTCACCGCTTAAACTGTCAGTGGAAGTGCGCAACCTTGAGCCAGGTTCCACTTACCGTTTCCGTGCCATTGCCATGAATAACTATGGTGAAAGCCCAGC
TAGCGCCACCTCCAGGCCATACCAGGTCTCCATGTCAAGTTCACCTGTCTCGAACCGTCCAGTCACTGGGCCTCACATCTCTTCCACTGATGCTGTTAGTGATACTCAGATTCTGTTACGCT
GG

h)CCAATAAGAATCCCTCCAAAGCTCCAATTGTGTCTCTACCAGAGAAAACTCCAGAAGATAAAACTACTAACACTTATTATGGAGTCGTCATACATGACAGAGTTCCAGAGGCTCCAGACC
GCCCCACAATCTCAACGGCGACAGAAAGTTCTGTTTACGTCACATGGATACCTCGGGCCAATGGTGGCTCTCCGATCACTGCATTTCGCGTGGAGTACAGAAAACAGGGCCGTAATGGAGAC
TGGATCATCGCAGCTGATAACATCTCACCGCTTAAACTGTTCCACTTACCGTTTCCGTGTCATTGCCATGAATAACTATGGTGAAAGCCCAGCTAGCGCCACCTCCAGGCCATACCAGGTCT
CCATGTCAAGTTCACCTGTCTCGAACCGTCCAGTCACTGGGCCTCACATCTCTTCCACTGATGCTGTTAGTGATACTCAGATTCTGTTACGCTGG

i)CCAATAAGAATCCCTCCAAAGCTCCAATTGTGTCTCTACCAGAGAAAACTCCAGAAGATAAAACTACTAACACTTATTATGGAGTCGTCATACATGACAGAGGTTCCACTTACCGTTTCC
GTGTCATTGCCATGAATGACTATGGTGAAAGCCCAGCTAGCGCCACCTCCAGGCCATACCAGGTCTCCATGTCAAGTTCACCTGTCTCGAACCGTCCAGTCACTGGGCCTCACATCTCTTCC
ACTGATGCTGTTAGTGATACTCAGATTCTGTTACGCTGG

Supplementary Figure 8. Sequences of the *cdon* products generated by injection of splicing MOs. After splicing MO injections embryos were collected and the generated Cdon products were amplified by RT-PCR. The obtained amplicons are shown in figure 4. The corresponding sequences are illustrated in (**a-i**) as follow: (**a-d)** sequences of bands 1-4 in Fig.4c; (**e, f)** sequences of bands 5 and 6 in Fig.4f; (**g-i)** sequences of bands 7-9 in Fig.4i. **a**) Wild type band. **b**) Cryptic splice site activation generated a deletion of 73pb, producing a frame-shift mutation. **c**) Cryptic splice site activation generates a deletion of 162pb that does not produce a frame-shift mutation. **d**) Deletion of Cdon exon 8 (284pb) produces a frame-shift mutation. **e**) Wild type band. **f**) Deletion of Cdon exon 14 (123pb) does not produce a frame-shift mutation. **g**) Wild type band. **h**) Cryptic splice site activation generates a deletion of 29pb, producing a frame-shift mutation. **i**) Deletion of Cdon exon 11 (207pb) does not produce a frame-shift mutation.

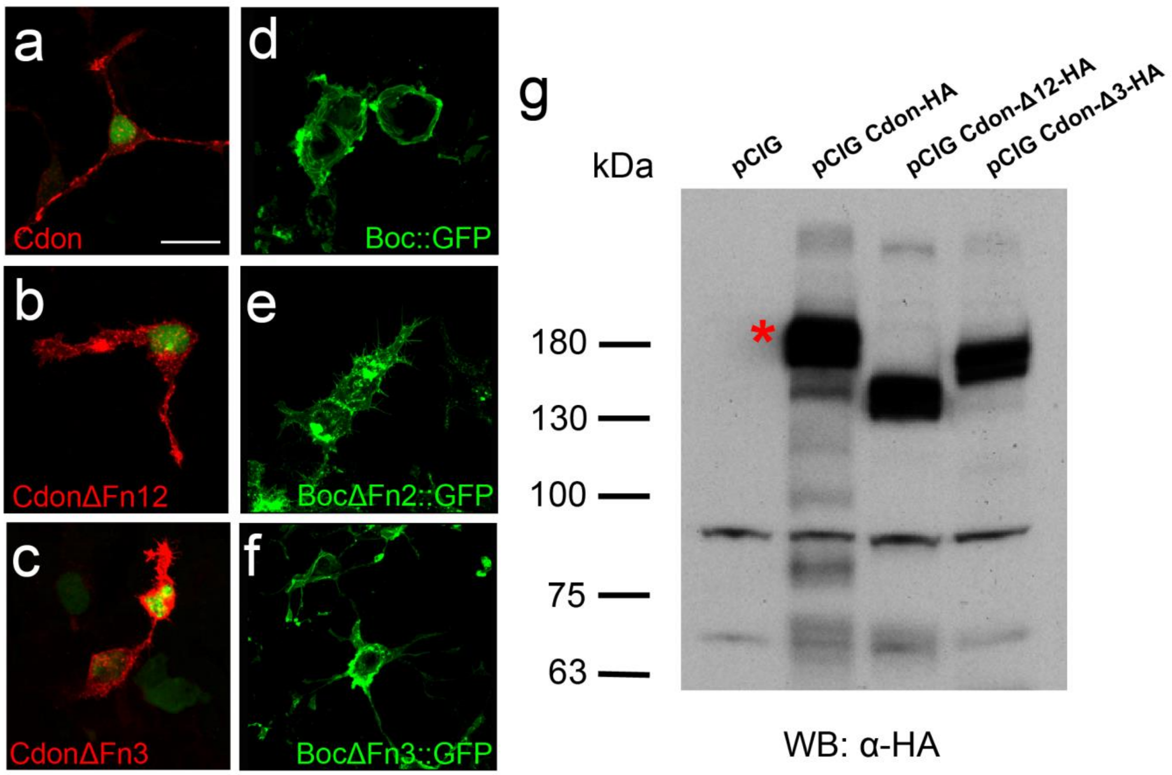

Supplementary Figure 9. Deletion of the fibronectin domains in Cdon and Boc does not affect protein stability or membrane localization. Fluorescent images of HEK cells transfected with Cdon, Boc-EGFP or their deleted versions as indicated in the panels (**a**-**f**). Cdon localization was determined by IHC. In all cases the fluorescent signal is detected at the plasma membrane. **g**) Western Blot analysis of lysates from cells transfected with Cdon or its derivatives shows that Cdon and deleted constructs are translated properly. The green signal in **a**-**c** corresponds to a nuclear GFP carried by pCIG vectors. The asterisk indicates the position of the band corresponding to full-length Cdon. Scale bar: 2 µm.

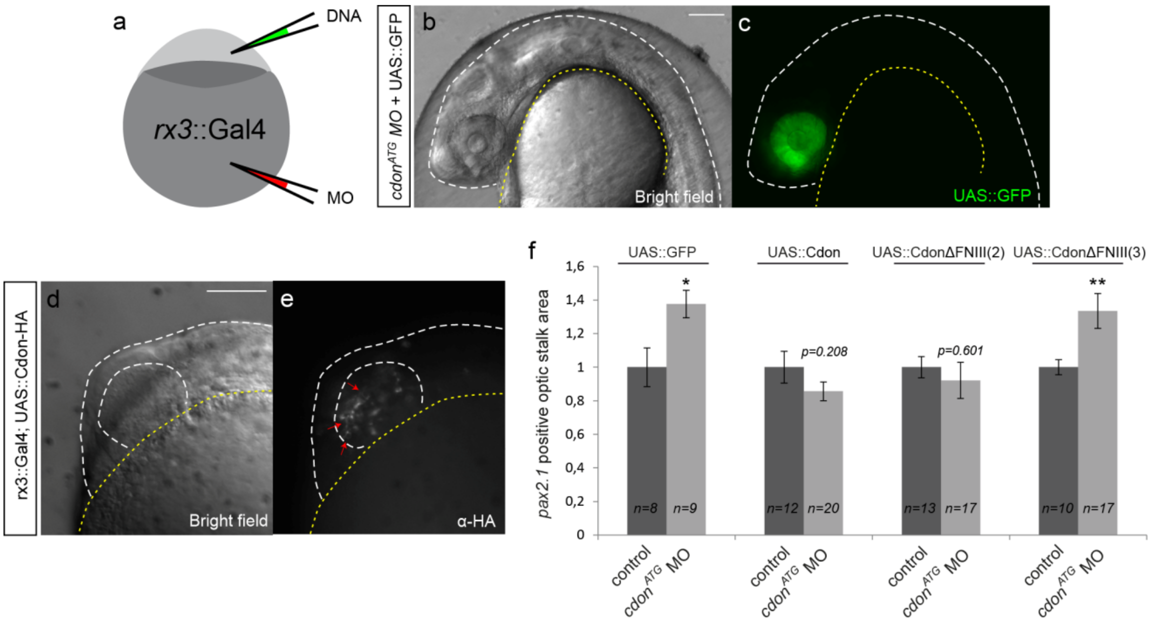

Supplementary Figure 10. Overexpression of *Cdon* or *CdonΔFnIII(2)* in the eye rescues the optic stalk phenotype of *cdon* morphants. a) The schema depicts the injection method used in rescue experiments. Embryos expressing Gal4 protein in the *rx3* positive domain (rx3::Gal4) were injected at 1-cell stage into the cell with UAS::GFP, UAS::Cdon, UAS::CdonΔFnIII(2) or UAS::CdonΔFnIII(3) DNA. After DNA injection, the eggs were injected with *Cdon*^{ATG} MO into the yolk and the embryos were let develop until 26hpf (**b**). **c**) GFP expression was detected in the retinal and hypothalamic domains of the injected embryos. α-HA immunostaining in *rx3::Gal4* embryos injected with UAS::Cdon DNA confirmed localized expression of Cdon in the retina of injected embryos (**d**, **e**). Note the membrane localization of the HA-signal (red arrows in **e**. **f**) Quantification of optic stalk *pax2.1*-positive domain in embryos co-injected with different UAS DNAs and *cdon*^{ATG} MO at 26-28hpf (*p<0.05, **p<0.01; Student's *t*-test). The number of embryos analysed in each case is indicated in each column and are as follow: control/UAS::GFP, n=8; *Cdon*^{ATG} MO/UAS::GFP, n=9; control/UAS::Cdon, n=12; *Cdon*^{ATG} MO/UAS::Cdon, n=20; control/UAS::CdonΔFnIII(2), n=13; *Cdon*^{ATG} MO/UAS::CdonΔFnIII(2), n=17; control/UAS::CdonΔFnIII(3), n=10; *Cdon*^{ATG} MO/UAS::CdonΔFnIII(3), n=17. Error bars represent s.e.m. Note that the expansion of the *pax2.1*-positive optic stalk domain generated by *cdon*^{ATG} MO injection was less evident than that reported in Fig. 4l, likely owning to MO injection at two-four cells' stage (see Methods). Scale bar: 100 µm.

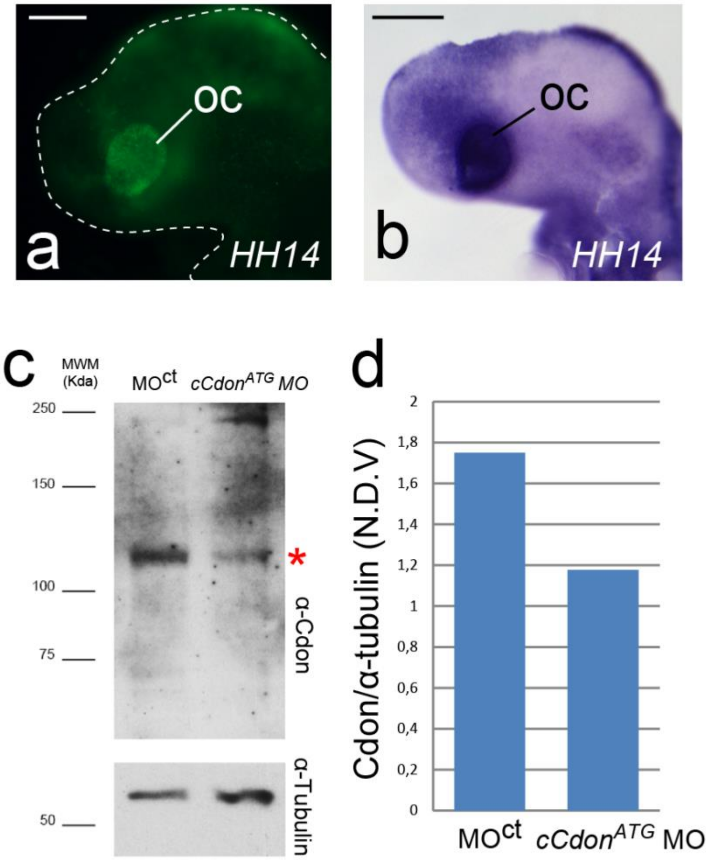

Supplementary Figure 11. ***cCdon**^{ATG}* **MO efficiently interfere with *Cdon* expression. a, b**) Carboxyfluorescein conjugated chick *Cdon* MO (*cCdon*^{ATG}) and control MO were targeted by electroporation in the optic region of HH8 chick embryos and analyzed at HH14 when Cdon is abundantly expressed in the eye (compare MO distribution in **a** with *in situ* signal in **b**). Targeted eyes were dissected and processed for WB analysis with antibodies against mouse Cdon normalized to α-tubulin levels (**c**). **c, d**) *cCdon*^{ATG} MO interferes with Cdon levels in the targeted eyes (n=8 electroporated eyes for each condition) (N.D.V, normalized density values). oc, optic cup. Scale bars: 200 µm.

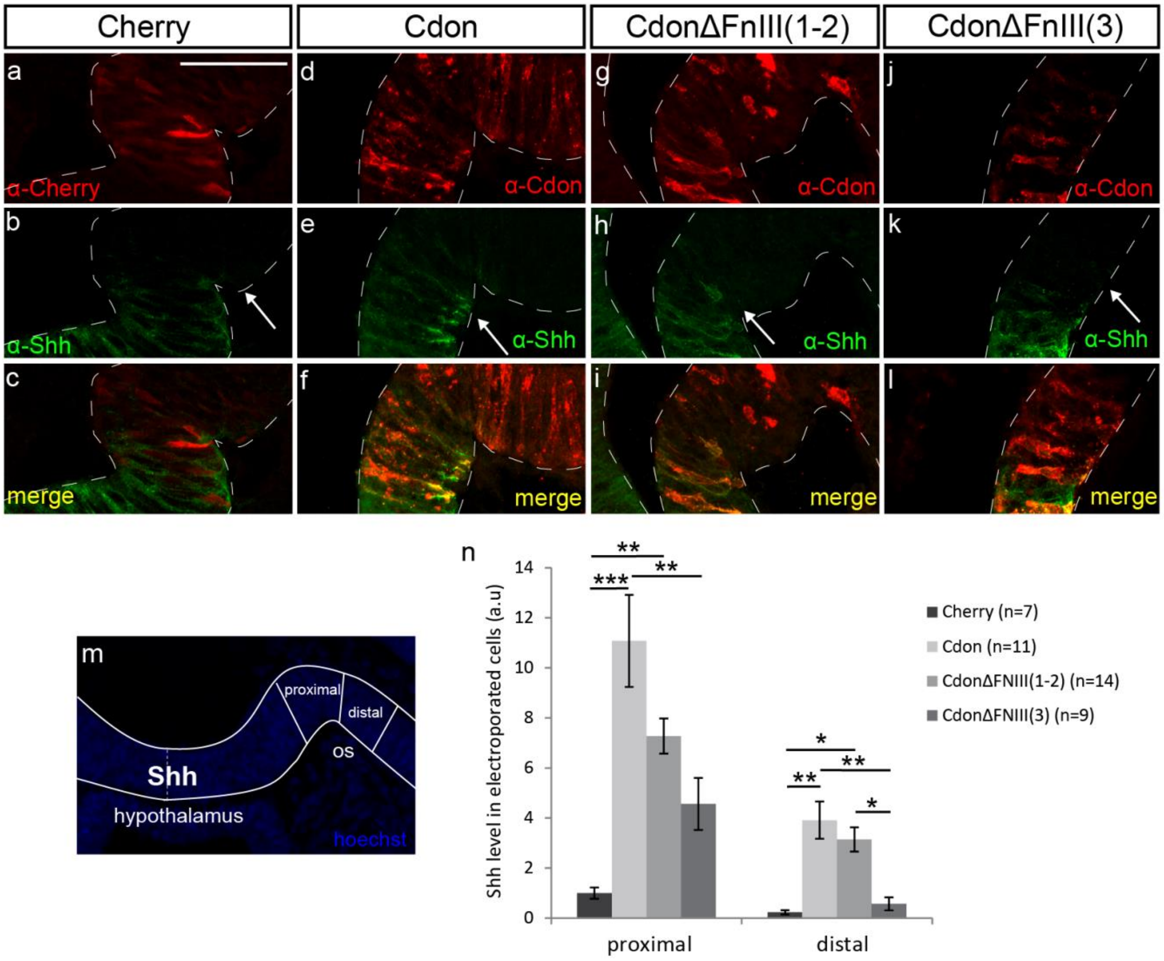

Supplementary Figure 12. Cdon overexpression modifies Shh protein distribution in the optic stalk neuroepithelium. a-l) Confocal analysis of coronal sections at the level of the optic vesicle of HH14 chick embryos electroporated at HH8 with a construct carrying mCherry (**a-c**), Cdon (**d-f**), or its deleted derivatives CdonΔFnIII(1-2) (**g-i**) and CdonΔFnIII(3) (**j-l**). Sections were immunostained with antibodies against mCherry (**a**), Cdon (**d, g, j**) and Shh (**b, e, h, k**). Shh protein localizes to Cdon expressing cells (**d-f**) in which it accumulates at the basal region of the neuroephitelium (**e, f** white arrow). A similar accumulation is observed in the presence of the CdonΔFnIII(1-2) construct (**g-i** white arrow) but was hardly detectable in mCherry or CdonΔFnIII(3) expressing cells (**b, c** and **j-l**). **m**) Coronal section of HH14 chicken embryo stained with Hoechst at the level of the optic vesicles to illustrate the areas selected to quantify Shh immunostaining in Cdon overexpressing cells. **n**) The graph represents the quantification of Shh accumulation in electroporated cells. *p<0.05, **p<0.01, ***p<0.001; Student's *t*-test. Note that there is no statistical difference in Shh levels of Cdon and CdonΔFnIII(1-2) overexpressing cells (proximal, *p*=0.074; distal, *p*=0.7). The number of quantified sections (1 section per embryo) is indicated in the graph labels. The number of analysed embryos is the following: Cherry, n=7; Cdon, n=11; CdonΔFnIII(1-2), n=14 and CdonΔFnIII(3), n=9. Error bars represent s.e.m. Scale bar: 50 µm.

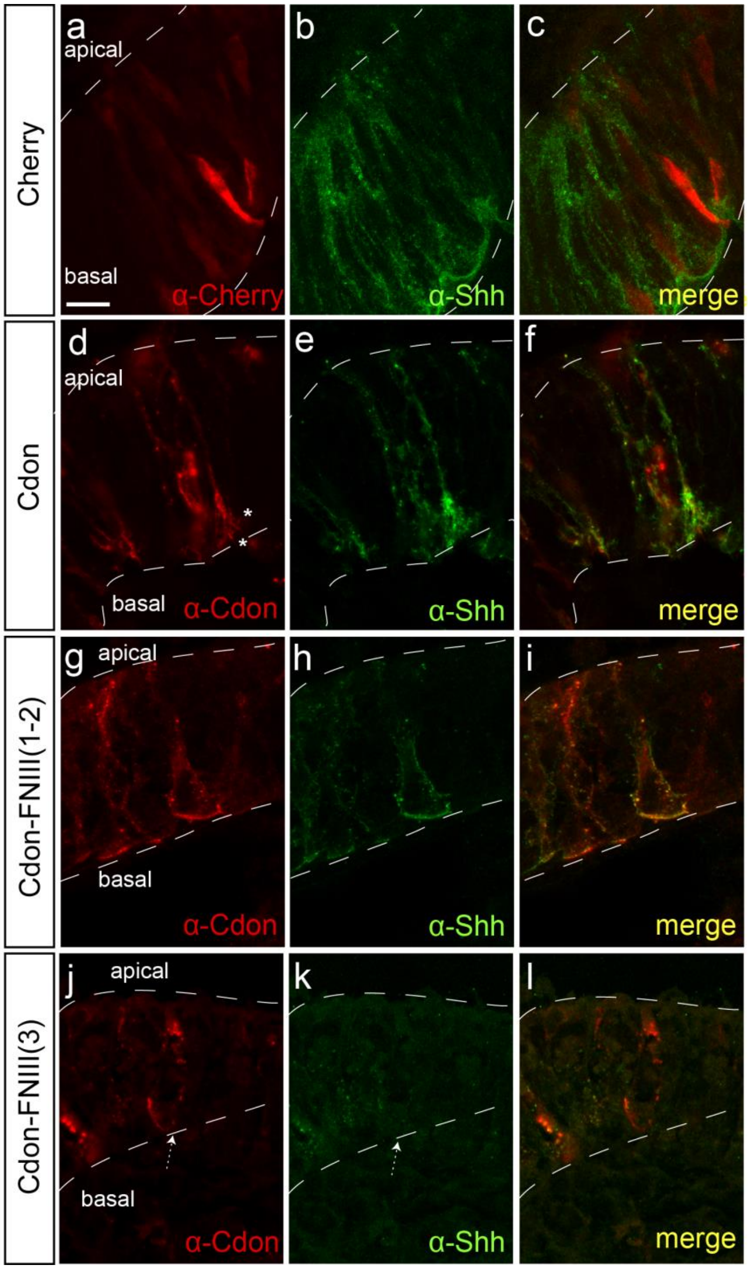

Supplementary Figure 13. Cdon promotes morphological changes of the basolateral end-foot of neuroepithelial cells. Confocal analysis of coronal sections at the level of HH14 chicken optic stalk electroporated at HH8 with a construct carrying mCherry (**a**-**c**) Cdon (**d-f**), or its deleted derivatives CdonΔFnIII(1-2) (**g-i**) and CdonΔFnIII(3) (**j-l**). Sections in (**a-l**) were immunostained with antibodies against Cherry (**a**) Cdon (**d, g, j**) and Shh (**b, e, h, k**). Note how cells electroporated with Cdon (**d**) or CdonΔFnIII(1-2) (**g**) present an enlarged basal end-foot when compared to mCherry or CdonΔFnIII(3) neuroepithelial cells (**a, j** arrows). This enlarged end-foot is a preferential site of Shh accumulation (**e, h**). Note the filopodial-like structures in the basal end-foot of Cdon expressing cell (**d**, asterisks). Scale bar: 10 μm.

Supplementary Table 1. List of the morpholinos used in this study

Name	Sequence (5´-3´)	Modification	Specificity	Working concentration
MO^{ct}	CCTCTTACCTCAGTTACAATTTATA	3´- Lissamine	-	160uM-200uM
$cdon^{ATG}$	ATAATCTCAGGCCACCGTCCTCCAT	3´- Lissamine	*Danio Rerio*	160uM
$cdon^{spl8}$	ACTGTATGAACTCTTCTCACCTTGC	-	*Danio Rerio*	200uM
$cdon^{spl11a}$	AGGAACTAGAGAATGAATCCAACCA	-	*Danio Rerio*	200uM
$cdon^{spl11d}$	GCAGTAACCTCACCTGGCTCAAGGT	-	*Danio Rerio*	200uM
$cdon^{spl14}$	GACGGTACTTCAGACTCTCACCTTT	-	*Danio Rerio*	400uM
cMO^{ct}	CCTCTTACCTCAGTTACAATTTATA	3´-Carboxyfluorescein	-	2mM
$cCdon^{ATG}$	CCGACTGCATAGCGCCCGGACAGCA	3´-Carboxyfluorescein	*Gallus gallus*	2mM

Note: **a** and **d** in $cdon^{spl11a}$ and $cdon^{spl11d}$ MO denote splice "acceptor" and "donor", respectively.

Supplementary References

1. Consortium, T.U. Reorganizing the protein space at the Universal Protein Resource (UniProt). *Nucleic Acids Res* **40**, D71-75 (2012).
2. Goujon, M. *et al.* A new bioinformatics analysis tools framework at EMBL-EBI. *Nucleic Acids Res* **38**, W695-699 (2010).
3. Sievers, F. *et al.* Fast, scalable generation of high-quality protein multiple sequence alignments using Clustal Omega. *Mol Syst Biol* **7**, 539 (2011).
4. Bae, G.U. *et al.* Mutations in CDON, Encoding a Hedgehog Receptor, Result in Holoprosencephaly and Defective Interactions with Other Hedgehog Receptors. *Am J Hum Genet* (2011).
5. McLellan, J.S. *et al.* The mode of Hedgehog binding to Ihog homologues is not conserved across different phyla. *Nature* **455**, 979-983 (2008).
